# Gut microbiota-host co-metabolism in hypertriglyceridemia-associated acute pancreatitis: from causality to precision intervention

**DOI:** 10.3389/fimmu.2026.1932634

**Published:** 2026-09-16

**Authors:** Jun Tian, Ke Chen, Lin Wang, Jing Wang

**Affiliations:** 1 Department of Gastroenterology, Songjiang Hospital Affiliated to Shanghai Jiaotong University School of Medicine, Shanghai, China; 2 Department of Gastroenterology, Shanghai Songjiang Clinical Medical College of Nanjing Medical University, Nanjing, China

**Keywords:** aryl hydrocarbon receptor, gut microbiota, host-microbiota co-metabolism, hypertriglyceridemia-associated acute pancreatitis, microbial metabolites, precision intervention, short-chain fatty acids

## Abstract

Clinical outcomes in hypertriglyceridemia-associated acute pancreatitis remain highly heterogeneous. Despite comparable lipid burdens, patients may experience anything from mild interstitial edema to fulminant necrotizing disease, a divergence that current models struggle to explain. Hypertriglyceridemia-associated acute pancreatitis (HTG-AP) now accounts for roughly one in nine acute pancreatitis cases worldwide, but serum triglyceride levels alone do not explain the significant East-West mortality gap (4.1% versus 1.0%). Emerging evidence shifts the focus away from the pancreas itself and toward a gut-pancreas co-metabolic circuit. Within this circuit, microbial metabolites engage host receptors and determine whether local injury escalates into systemic disease. In this review, we examine four interconnected axes that form The mechanistic backbone of this circuit: LPS-TLR4-lysophosphatidylcholine, short-chain fatty acid-GPR43/HDAC, tryptophan-aryl hydrocarbon receptor, and bile acid-FXR/TGR5—recognizing that direct HTG-AP-specific evidence is currently strongest for the LPS-TLR4-LPC axis, while the other three axes are supported by evidence from AP broadly and await HTG-AP-specific validation. We critically examine the cross-talk among these pathways and apply the Bradford Hill criteria to assess the validity of current causal inferences in the gut-pancreas axis. From a translational perspective, we introduce the concept of “functional metabolite guilds” (metabolite clusters sharing protective endpoints) to inform and streamline future postbiotic formulation designs. We also outline a co-metabolic stratification logic to guide patient selection in future trials. The ultimate goal is to move beyond descriptive microbiome catalogs toward precision interventions that match metabolic deficits with guild-based restoration strategies.

## 1 Introduction

### 1.1 Shifting etiological landscape of acute pancreatitis

Acute pancreatitis (AP) is one of the most common gastrointestinal emergencies. Its annual incidence now exceeds 34 per 100,000 population (1). Gallstones and alcohol have long dominated the causes, but hypertriglyceridemia (HTG), defined as serum triglycerides above 500 mg/dL and conferring severe risk above 1,000 mg/dL, has overtaken alcohol as the second leading cause in China, accounting for about 20% of cases. The same trend is emerging globally (2, 3). A 2025 meta-analysis of 110 studies (n = 3,057,428) estimated the global proportion of HTG-AP at 11.6%, but this figure hides a stark geographic divide: 16.3% in Eastern countries versus 5.4% in Western countries (4). Mortality in Eastern cohorts reached 4.1%, four times the 1.0% seen in Western settings. A Chinese multi-center cohort (n = 3,224) found that HTG-AP carried a nearly three-fold higher mortality risk than AP from other causes (OR 2.779, 95% CI 1.60–4.81) (4).

This persistent mortality gap does not correlate with baseline clinical severity or initial ICU resource allocation. That strongly suggests that post-admission management and possibly population-specific host-microbiota interactions matter more than how sick the patient looks on arrival. A cross-population metagenomic comparison of over 2,000 healthy adults from six countries revealed consistent differences in gut microbial species and pathways between Western and non-Western populations. China showed a unique profile that partly mirrors the Westernization of dietary patterns (5). Even within China, the Guangdong Gut Microbiome Project documented substantial regional variation in genera such as Desulfovibrio and Faecalibacterium (6), underscoring that microbiome-based diagnostics cannot be simply extrapolated from one population to another.

A parallel systematic review of 56,617 AP cases found that HTG-AP patients were younger (mean age 41.5 vs. 50.3 years) and predominantly male (68.7% vs. 57.3%) (7, 8). Temporal trends from 2002 to 2023 show that while global HTG-AP prevalence remained relatively stable, China experienced a significant upward trajectory (P = 0.0119), likely driven by dietary Westernization (8). Clinically, HTG-AP carries a higher burden of acute respiratory distress syndrome, acute kidney injury, and recurrence (9). Yet the therapeutic toolkit remains largely supportive. Common triglyceride-lowering measures such as fasting, insulin, heparin, and plasma exchange have been increasingly questioned for their ability to alter the disease course (10), pointing to an incomplete mechanistic understanding of what drives progression.

### 1.2 Limitations of the classical lipotoxicity model

The prevailing framework holds that circulating triglycerides, upon reaching the pancreatic microvasculature, are hydrolyzed by pancreatic lipase to release excessive free fatty acids. These then trigger intracellular acidosis, calcium overload, mitochondrial failure, and acinar cell necrosis (11). While supported by robust *in vitro* and animal data, classical lipotoxicity alone fails to fully capture the diverse phenotypic spectrum seen in clinical practice. Patients with comparable triglyceride levels often diverge dramatically: some show only mild interstitial edema, while others spiral into necrotizing pancreatitis with multi-organ dysfunction. This dissociation between circulating triglyceride levels and clinical outcomes indicates that the classical lipotoxicity framework requires further conceptual expansion. It fundamentally overlooks the extra-pancreatic modifiers — specifically the gut-derived metabolic inputs — that can turn a localized lipid insult into a systemic disease (12).

### 1.3 Causal evidence for the gut-pancreas axis

The gastrointestinal tract has long been recognized as the “motor” of multi-organ failure in severe AP, with intestinal barrier disruption, bacterial translocation, and endotoxemia amplifying systemic inflammation (13). For many years, changes in the gut microbiota were viewed as passive consequences of pancreatic inflammation. Over the past five years, three complementary lines of causal evidence have overturned that view.

First, gnotobiotic experiments have shown that fecal microbiota transplantation from diseased donors transfers susceptibility (14). Second, Mendelian randomization (MR) studies, which use germline genetic variants as instrumental variables, have established that gut microbiota composition causally influences AP risk, independent of confounding by the disease state (15, 16). Third, intervention studies in animal models have demonstrated that targeted microbiota modulation reduces AP severity through defined molecular pathways (17, 18). A 2025 MR meta-analysis integrating ten studies from European and East Asian populations confirmed consistent causal signals: for instance, Odoribacter emerged as protective, while Olsenella and Clostridium leptum conferred risk (19). A separate bidirectional MR study with mediation analysis, spanning 473 gut microbiota taxa, 233 metabolites, and 731 immune cell traits, revealed that circulating metabolites — not the immune cells themselves — are the key mediators of microbiota effects on pancreatitis, with 24 distinct mediation pathways identified (16).

HTG-AP provides a highly relevant model for investigating this bidirectional causality. The lipid-rich environment itself acts as a selective pressure on the gut microbial ecosystem. Hypertriglyceridemia remodels the community composition, and the resulting dysbiosis simultaneously injures the intestinal barrier and fuels systemic inflammation. This bidirectional relationship, in which HTG is both a cause and a consequence of microbial imbalance, makes HTG-AP a particularly tractable setting for testing causal inference methods and for designing microbiome-targeted interventions.

### 1.4 A co-metabolic perspective on HTG-AP

Host-microbiota crosstalk is primarily mediated by microbial metabolites—small molecules derived from microbial enzymatic activity that engage host receptors to reprogram metabolic pathways. When this chemical conversation breaks down, as it does in HTG-AP, the consequences reverberate from the intestinal epithelium to the pancreatic acinus and beyond. Accumulating evidence indicates that HTG-AP severity depends on the equilibrium between pathogenic co-metabolic signals (e.g., the LPS-TLR4-lysophosphatidylcholine cascade) and protective pathways carried by short-chain fatty acids, tryptophan-derived AhR ligands, and secondary bile acids.

Previous reviews have catalogued taxonomic shifts or examined host metabolism in isolation (20, 21). What has been missing is an integrated framework that treats these metabolite axes as a functionally coupled network. To move beyond descriptive correlations, this review evaluates the causal evidence for these co-metabolic axes using the Bradford Hill framework. Building on this, we propose the concept of “functional metabolite guilds”—operationally defined as clusters of microbial-derived metabolites that converge on a shared host-protective endpoint (e.g., barrier fortification, NLRP3 inflammasome suppression, or regulatory T cell differentiation), irrespective of their chemical scaffolds, phylogenetic origins, or upstream biosynthetic pathways-. Guild membership is determined by functional convergence at the host response level—the “last common pathway” through which structurally distinct metabolites exert equivalent protective effects—rather than by structural similarity, taxonomic source, or receptor identity. This concept is proposed as a heuristic to guide postbiotic formulation design, not as an established therapeutic principle; its validity requires prospective experimental testing.

### 1.5 Literature search and evidence appraisal

This narrative review was informed by a systematic literature search of PubMed, Web of Science, and the Cochrane Library for publications up to June 2026, using the following search strategy: (“hypertriglyceridemia” OR “hypertriglyceridemic” OR “HTG”) AND (“acute pancreatitis” OR “pancreatitis”) AND (“gut microbiota” OR “microbiome” OR “microbial metabolite” OR “short-chain fatty acid” OR “tryptophan” OR “bile acid” OR “LPS” OR “TLR4”). Additional searches were conducted for specific intervention modalities: (“probiotic” OR “postbiotic” OR “fecal microbiota transplantation” OR “prebiotic”) AND (“acute pancreatitis” OR “pancreatitis”). Reference lists of retrieved articles and relevant review papers were hand-searched for additional citations. No language restrictions were applied.

To construct **Table 1**, we classified the evidence for each intervention or pathway into three tiers using the following operational rules:

**Table 1:** Evidence hierarchy of gut–pancreas co-metabolic axes and microbiota-targeted interventions in HTG-AP.

Category	HTG-AP Evidence	Specific mechanism/intervention	Confirmed (animal/human)	Hypothesized (indirect evidence)	Conceptual (awaiting validation)
LPS-TLR4-LPC axis	Direct (14)	HTG -gut dysbiosis -LPS translocation -TLR4 activation -LPC generation -pancreatic injury	Mouse: FMT transfers susceptibility, Tlr4 KO abrogates effect (Song et al., 2025) Human: MR studies support causality (He et al., 2024; Zeng et al., 2025)	–	TLR4 antagonist efficacy in acute HTG-AP
SCFA-GPR43/HDAC axis	AP broadly (22)	Acetate -GPR43 -suppresses M1 macrophage polarization	Mouse: *B. pseudolongum*-derived acetate; protection lost in Gpr43 KO (Guan et al., 2026)	–	–
	AP broadly (23)	Butyrate- HDAC inhibition -epigenetic regulation of anti-inflammatory genes	Mouse: inulin-derived SCFAs mimicked by HDAC3 inhibitor (Li et al., 2024)	Synergy between GPR43 and HDAC pathways in AP	Optimal dosing and timing of butyrate in HTG-AP patients
		Serum SCFA alterations associate with AP	Human: serum acetate significantly elevated in AP vs controls (P < 0.001) (Yazici et al., 2023)	–	–
Trp-AhR axis	AP broadly (24)	*Lactobacillus*-derived norharman -HDAC inhibition -Rftn1 upregulation -suppresses M1 polarization	Mouse: norharman supplementation protective; myeloid-specific Rftn1 KO abrogates effect (Zhou et al., 2023)	–	Synbiotic strategy (tryptophan + AhR ligand-producing *Lactobacillus*) in humans
	Direct (18)	Rhubarb polysaccharide enriches *Lactobacillus*/*Akkermansia* -modulates tryptophan metabolism	Mouse: multi-omics integration (Chen et al., 2025)	–	–
		Butyrate -HDAC inhibition -upregulates AhR expression	–	HDAC inhibitors upregulate AhR in non-AP models; not tested in AP	Direct molecular link between butyrate and AhR in HTG-AP
BA-FXR/ TGR5 axis	AP broadly (25–27)	Secondary bile acids -FXR/TGR5 activation- suppresses NF-κB/NLRP3	Mouse: conjugated bile acids alleviate AP via TGR5/NLRP3 (Zhang et al., 2024) Mouse: *Odoribacter laneus* protects via BA-FXR (Gu et al., 2026)	–	–
		HTG -altered primary BA pool -suppresses BSH-active bacteria-reduced secondary BAs	–	Supported by HTG mouse models and functional prediction (Song et al., 2026)	Direct validation in HTG-AP patients
Probiotics	Direct (28)	*Akkermansia muciniphila* (mucus restoration)	Mouse: attenuates HTG-AP severity (Yang et al., 2025) Registered trial (no results yet)— ChiCTR2500112068; status: recruiting; accessed 2026-06-15	–	Safety and efficacy when administered in acute phase (<48h)
	AP broadly (29)	*Faecalibacterium prausnitzii* (butyrate producer)	Mouse: alleviates recurrent AP via oleic acid/MAPK/NF-κB (Qin et al., 2025)	–	–
	AP broadly (22)	*Bifidobacterium pseudolongum*(acetate-GPR43)	Mouse: preventive administration effective (Guan et al., 2026)	–	Post-onset administration efficacy
		*Lactobacillus* spp. (multi-guild: SCFA+AhR ligands)	Mouse: *L. paracasei* LP18 (Cao et al., 2026) Registered trial (no results yet) — ChiCTR2500108211; status: recruiting; accessed 2026-06-15	–	–
Postbiotics	AP broadly (30)	SCFA mixture (oral/colon-targeted)	Mouse: inulin-derived SCFAs protective via HDAC3 (Li et al., 2024) Registered trial (no results yet) — NCT06147635; status: recruiting; accessed 2024-06-17	–	Feasibility of colonic delivery in ICU setting (ileus, high intra-abdominal pressure)
		Tributyrin (butyrate pro-drug) Pickering emulsions	Effective in colitis model (Sun et al., 2025)	–	Validation in HTG-AP models and patients
	AP broadly (24)	Norharman	Mouse: supplementation attenuates AP (Zhou et al., 2023)	–	–
		Secondary bile acids (LCA/DCA)	Mouse: direct supplementation protective (Wan et al., 2020)	–	Optimal dosing and safety in HTG-AP
Natural polysaccharides	Direct (18)	Rhubarb polysaccharide	Mouse: modulates tryptophan metabolism, enriches protective taxa (Chen et al., 2025)	–	Validation in HTG-AP patients
	AP broadly (23)	Inulin	Mouse: protective via SCFA-HDAC3 (Li et al., 2024)	–	–
FMT/defined consortia	Direct (14)	Fecal microbiota transplantation (FMT)	Mouse: HTG-AP microbiota transfers susceptibility (Song et al., 2025) Registered trial (no results yet) — ChiCTR2500099245; status: Not yet recruited; accessed 2026-06-15	–	Standardized donor screening, safety, interaction with antibiotics
	Proposed	Defined microbial consortium (SCFA producers+mucus restorer+ tryptophan metabolizers)	–	–	Conceptual framework; no experimental validation yet

“Direct” — evidence generated directly in HTG-AP models or patient cohorts;.

“AP broadly” — evidence derived from non-HTG AP models or mixed-etiology human cohorts;.

“Proposed” — conceptual proposals lacking experimental validation;

AhR, aryl hydrocarbon receptor; AP, acute pancreatitis; APACHE II, Acute Physiology and Chronic Health Evaluation II; BA, bile acid; BISAP, Bedside Index for Severity in Acute Pancreatitis; BSH, bile salt hydrolase; DCA, deoxycholic acid; EEN, early enteral nutrition; FMT, fecal microbiota transplantation; FXR, farnesoid X receptor; HDAC, histone deacetylase; HTG-AP, hypertriglyceridemic acute pancreatitis; ICU, intensive care unit; KO, knockout; LCA, lithocholic acid; LPC, lysophosphatidylcholine; LPS, lipopolysaccharide; MAPK, mitogen-activated protein kinase; MR, Mendelian randomization; NF-κB, nuclear factor kappa B; NLRP3, NOD-like receptor protein 3; SCFA, short-chain fatty acid; TGR5, Takeda G-protein-coupled receptor 5; TLR4, Toll-like receptor 4; WT, wild-type.

(A) “Confirmed” evidence denotes mechanistic pathways or clinical trial registrations directly validated within the specific context of acute pancreatitis models or human cohorts; “Hypothesized” evidence indicates biological actions validated in non-pancreatitis models (e.g., colitis, metabolic syndrome) that possess strong physiological plausibility for HTG-AP translation; “Conceptual” positions represent theoretical frameworks currently awaiting direct experimental validation.

(B) Mouse study citations (e.g., Song et al., 2025; Guan et al., 2026) emphasize distinct therapeutic windows; preventive models imply administration prior to AP induction, whereas post-onset models denote intervention applied after the establishment of pancreatic acinar necrosis.

(C) Clinical trial identifiers prefixed with “ChiCTR” refer to registrations within the Chinese Clinical Trial Registry; identifiers prefixed with “NCT” refer to the U.S. National Institutes of Health ClinicalTrials.gov registry.

(D) Functional Metabolite Guilds are operationally defined herein as clusters of metabolically active microbial nodes that exploit distinct chemical substrates but converge synergistically on shared host-protective endpoints (e.g., paracellular tight junction fortification, inflammatory cellular de-priming), independent of their baseline phylogenetic relatedness.

“Confirmed (animal/human)” — findings supported by at least one *in vivo* study (animal model or human cohort) demonstrating a direct effect on AP-related outcomes, with the study design appropriate to the claim (e.g., intervention with control, genetic knockout, or FMT). For human data, we required published peer-reviewed results; for animal data, we required demonstrated effects on pancreatic injury, systemic inflammation, or survival.

“Hypothesized (indirect evidence)” — mechanisms or relationships that are biologically plausible and supported by evidence from non-pancreatic disease models (e.g., colitis, metabolic syndrome) or by *in vitro* mechanistic studies, but have not yet been tested in AP or HTG-AP models.

“Conceptual (awaiting validation)” — theoretical frameworks or proposed strategies that lack direct experimental support in any disease model and remain at the level of hypothesis generation.

Tier assignments were made by two authors independently (J.T. and K.C.), with disagreements resolved through discussion and, when necessary, adjudication by a third author (J.W.). The evidence context for HTG-AP specificity was further annotated in the “HTG-AP Evidence” column of **Table 1** based on the criteria defined in Section 2.3.

In this review, we synthesize current evidence on HTG-AP pathogenesis, focusing on the gut-pancreas co-metabolic circuit. We outline specific microbial metabolite axes, evaluate causal relationships using Bradford Hill criteria, and propose precision intervention strategies for future research.

## 2 Gut microbial dysbiosis in HTG-AP: from community shift to barrier failure

While hypertriglyceridemia initiates the local insult, the gut microbiota significantly modulates the downstream clinical trajectory. The interplay between circulating lipids and gut microbes exhibits bidirectional causality. HTG remodels the intestinal luminal environment, and the resulting dysbiosis determines, at least in part, whether pancreatic injury remains contained or escalates into systemic disease.

### 2.1 HTG-induced permissive microbial milieu

Elevated luminal lipid content does more than provide substrate for pancreatic lipase. It fundamentally changes the chemical environment in which gut microbes live. Triglyceride-rich chyme alters bile acid secretion patterns, changes the redox potential at the mucosal surface, modifies the pool of fermentable substrates, and exerts direct bacteriostatic or bactericidal effects on certain bacterial phylotypes through lipid-mediated membrane disruption (11). In this sense, HTG acts as a potent environmental filter that selectively reshapes the gut microbial community, suppressing some symbionts while allowing others to expand.

Once this HTG-sculpted dysbiosis takes hold, it feeds back on the host. The altered community compromises the intestinal barrier through multiple mechanisms: it erodes the mucus layer, weakens tight junctions, and suppresses antimicrobial peptide production. These changes allow bacterial structural components, most notably lipopolysaccharide (LPS), to translocate into the portal and systemic circulation. This phenomenon, often called metabolic endotoxemia, sets up a self-amplifying loop. Barrier injury permits endotoxin entry, endotoxemia fuels systemic inflammation, and systemic inflammation further disrupts barrier integrity (13).

We therefore view HTG as the “first hit” — it creates a permissive gut ecosystem. The consequent microbial dysbiosis is the “second hit” that determines whether acute pancreatitis tips into a severe, multi-organ phenotype. This two-hit logic helps resolve the clinical paradox: equally elevated triglycerides do not equally predict severe disease. The composition and functional capacity of an individual’s gut microbiota likely modulate the threshold at which local pancreatic injury becomes systemic (12, 31).

### 2.2 Conserved dysbiotic patterns across models

Despite inevitable variation in which specific genera are reported across studies, several conserved signatures recur in human cohorts and animal models. At the functional level, these boil down to a dual pathology: loss of short-chain fatty acid (SCFA)-producing capacity and gain of barrier-disrupting, pro-inflammatory activities.

Depletion of SCFA producers, most consistently Faecalibacterium prausnitzii, Lactobacillus species, and Odoribacter, has been documented in HTG-AP patients and is associated with disease severity, organ failure, and mortality (31). Parallel to this collapse of symbionts, an expansion of mucolytic and pro-inflammatory pathobionts, including Escherichia-Shigella, Enterococcus, and Desulfovibrio, has been repeatedly observed (13, 31). A particularly instructive example comes from Yang et al. (2025), who provided direct causal evidence in HTG-AP mouse models. The abnormal enrichment of Allobaculum mucilyticum directly aggravated oxidative stress-associated intestinal barrier dysfunction, facilitating bacterial translocation to the pancreas. In contrast, supplementation with the mucin-restoring symbiont Akkermansia muciniphila alleviated disease severity by preserving the mucus layer and dampening pro-inflammatory macrophage polarization in the gut (28). These reciprocal experiments demonstrate that specific microbial taxa can push the host-microbiota equilibrium in opposite directions.

### 2.3 Etiology-specific microbial signatures in HTG-AP

A question that remains underexplored is whether HTG-AP carries a microbial signature distinct from AP of other etiologies, or whether the observed dysbiosis is merely a generic response to pancreatic inflammation. Early 16S rRNA gene amplicon sequencing comparing biliary AP, hyperlipidemic AP (HLAP), and healthy controls suggested that HLAP indeed displays more pronounced dysbiosis and stronger functional impairment than biliary AP (32). A subsequent study focusing on gut microbiota-based biomarkers for AP subtype classification further supported the existence of etiology-specific signatures (33). In the clinical setting, gut microbiota composition at hospital admission has been shown to associate with subsequent disease severity and prognosis in HTG-AP patients (31). A European multi-center study recently demonstrated that admission microbiota profiles can predict post-discharge mortality, recurrent pancreatitis, and incident diabetes mellitus (34). Whether these signatures are sufficiently etiology-specific to serve as diagnostic or prognostic biomarkers, as opposed to severity markers common to all forms of AP, remains an open question. A caveat to interpreting human microbiome data in HTG-AP is confounding by comorbidities that cluster with hypertriglyceridemia—obesity, insulin resistance/diabetes, and alcohol exposure—each of which independently reshapes the gut microbiota and predicts AP severity. Thus, the dysbiosis signature attributed to HTG in observational studies may partly reflect these co-occurring metabolic conditions. MR does not fully resolve this, as its instruments target taxon abundance rather than metabolic context, and horizontal pleiotropy through adiposity is a likely violation. Diet-induced HTG animal models are similarly confounded, as they are simultaneously obesity models. To separate HTG effects from its correlates, future studies should consider: (1) BMI- and HbA1c-matched cohort analyses; (2) within-person longitudinal sampling across an HTG flare; and (3) mediation analyses using measured (rather than genetically proxied) confounders. Until such studies are performed, the HTG-specificity of reported dysbiosis signatures remains provisional. To contextualize the mechanistic evidence that follows, we note that—among the studies cited in this review—direct HTG-AP-specific evidence (defined as data generated in HTG-AP patient cohorts or in animal models where hypertriglyceridemia is the inducing or conditioning factor) is available for the LPS-TLR4-LPC axis (14), rhubarb polysaccharide intervention (18), Allobaculum mucilyticum/Akkermansia muciniphila studies (28), and Paneth cell studies (35). All other mechanistic evidence discussed below derives from AP broadly (non-HTG models or mixed-etiology human cohorts) and, while mechanistically informative, requires direct HTG-AP-specific confirmation.

### 2.4 Intestinal barrier failure as the final common pathway

Whatever the initial trigger, the unifying consequence of HTG-associated gut dysbiosis is disruption of the multi-layered intestinal barrier. The mucosal defense system consists of a physical mucus layer, paracellular tight junction proteins, chemical antimicrobial peptides, and an underlying immunological firewall (13). HTG-AP-associated dysbiosis compromises every layer. Mucolytic bacteria such as A. mucilyticum erode the protective mucus (28). Depletion of butyrate-producing bacteria starves colonocytes of their primary energy source and weakens tight junction assembly (17). Translocation of LPS and other pathogen-associated molecular patterns across the breached epithelium directly activates submucosal immune cells.

Paneth cells, located at the base of small intestinal crypts, have emerged as critical sentinels in this process. In a rat model of HTG-related acute necrotizing pancreatitis, gut dysbiosis was accompanied by decreased expression of α-defensin 5 and lysozyme in Paneth cells (35). Fu et al. (2022) subsequently showed that genetic ablation of Paneth cell function exacerbated gut dysbiosis, increased bacterial translocation, and worsened pancreatic injury, formally establishing these cells as gatekeepers at the gut-pancreas interface (36).

Consequently, HTG acts as the primary catalyst that erodes the microbial ecosystem necessary for maintaining barrier competence. The loss of this ecosystem function converts the gut from a bystander into an amplifier of pancreatic injury.

## 3 The core co-metabolic axes: how microbial metabolites shape HTG-AP severity

Before dissecting each axis, we emphasize that the strength of evidence varies along two dimensions: disease specificity (HTG-AP vs. AP broadly) and experimental system (human clinical data vs. animal models vs. mechanistic studies in non-pancreatic systems). Throughout this section, we explicitly identify the evidence context for each key claim. Where data specific to HTG-AP are limited, we clearly indicate extrapolation from broader AP models or from mechanistic studies in other inflammatory conditions.

Mechanistically, the gut-pancreas axis relies less on direct microbial translocation and more on the systemic dissemination of microbial metabolites. These small molecules bridge the disrupted intestinal ecosystem and the pancreas, enter the portal and systemic circulation, and engage host receptors to either amplify or restrain inflammation.

In HTG-AP, the net clinical trajectory can be understood as a balance between two opposing co-metabolic forces. Gut-derived lipopolysaccharide (LPS) acts as a primary pathogenic driver by reprogramming host lipid metabolism to exacerbate pancreatic damage. Conversely, an array of microbial metabolites—including short-chain fatty acids (SCFAs), tryptophan-derived AhR ligands, and secondary bile acids—ordinarily function to maintain barrier competence and restrict innate immune responses. All three are progressively lost as dysbiosis deepens. Critically, these four axes do not operate in parallel. They intersect, modulate one another’s signal strength, and together form an integrated network. The net output of this network likely determines whether a patient follows a mild or severe course. This section dissects the molecular logic of each axis while keeping their cross-talk in view (**Figure 1**).

**Figure 1:**
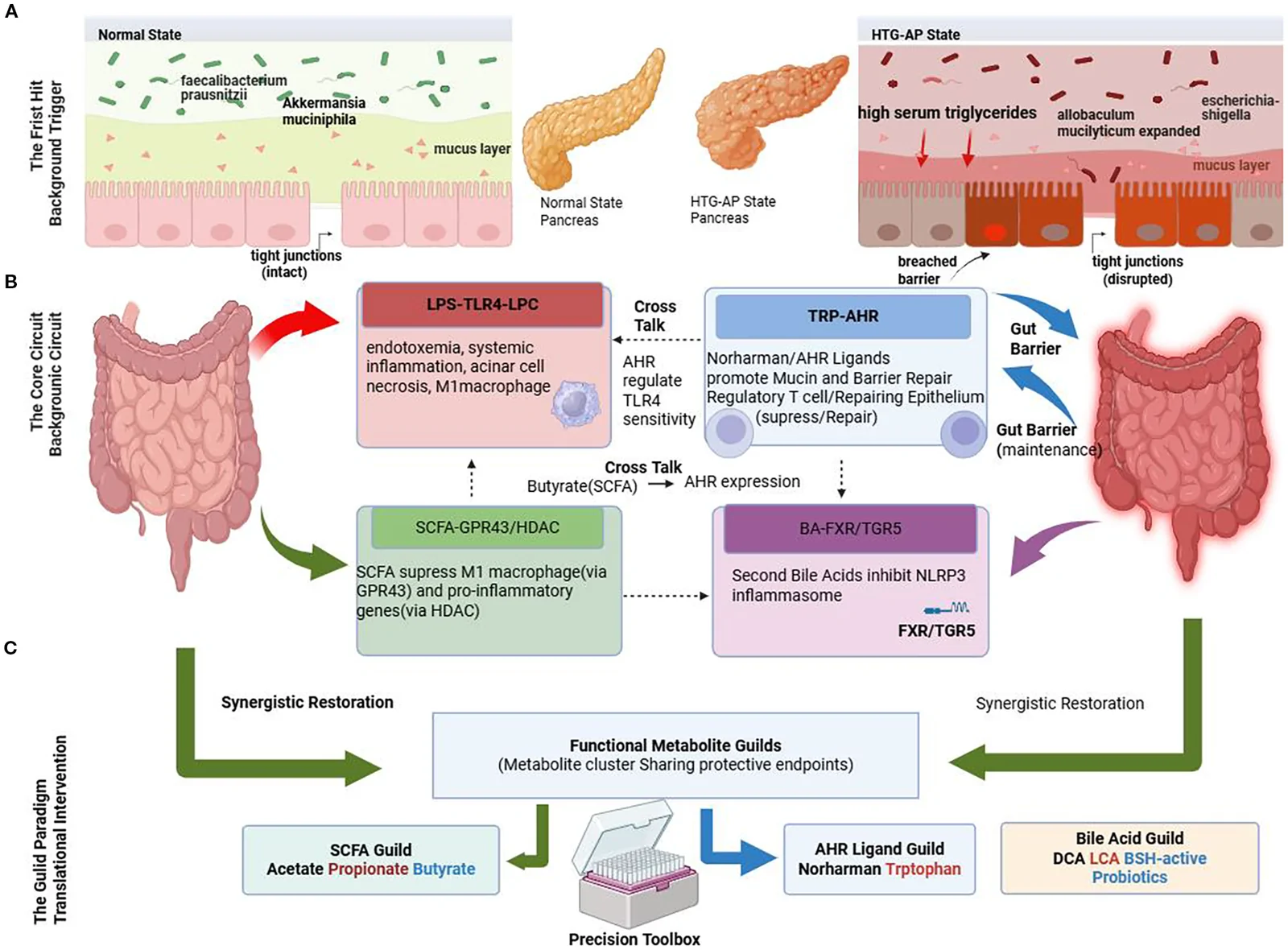
The gut-pancreas co-metabolic circuit in hypertriglyceridemia-associated acute pancreatitis (HTG-AP). **(A)** The “First Hit” and Background Trigger: Under normal physiological states, a dense mucus layer, intact tight junctions, and a highly diverse microbiota (characterized by symbiotic consortia such as Faecalibacterium prausnitzii and Akkermansia muciniphila) maintain target organ homeostasis. In the hypertriglyceridemic (HTG) state, a lipotoxic intraluminal milieu creates a selective environmental filter that causes a dysbiotic collapse, marked by the expansion of mucolytic and pro-inflammatory pathobionts (e.g., Allobaculum mucilyticum, Escherichia-Shigella), eroding the mucus barrier and breaking paracellular tight junctions. **(B)** The Core Circuit and Network Cross-Talk: Host-microbiota crosstalk is distributed across four coupled co-metabolic axes. The LPS-TLR4-LPC axis (red) represents the primary pathogenic branch, where translocated lipopolysaccharide (LPS) triggers Toll-like receptor 4 (TLR4) signaling to drive host glycerophospholipid rewiring and lysophosphatidylcholine (LPC) generation, inducing acinar cell necrosis and systemic endotoxemia. This damaging cascade is counter-regulated by three protective axes: the SCFA-GPR43/HDAC axis (green), which suppresses M1 macrophage polarization and epigenetically dampens pro-inflammatory transcription via histone deacetylase (HDAC) inhibition; the Trp-AhR axis (blue), where Lactobacillus-derived norharman/AhR ligands promote mucin expression and shift immune profiles toward regulatory T cell/repairing epithelium phenotypes; and the BA-FXR/TGR5 axis (purple), where secondary bile acids restrain NLRP3 inflammasome activation. Key network cross-talk interfaces include butyrate-mediated upregulation of AhR expression and AhR-driven modulation of host TLR4 sensitivity. **(C)** Conceptual Framework: The ‘Guild’ Paradigm for Translational Intervention (Proposed): Upstream replenishment via functional metabolite guilds (clusters of structurally distinct microbial metabolites sharing protective endpoints, including the SCFA, AhR Ligand, and Bile Acid guilds) is proposed to break the self-amplifying loop of barrier breakdown through synergistic restoration of the host gut firewall. Panel C depicts a conceptual proposal for guild-based intervention strategies. The guild framework is defined by convergence on shared host-protective endpoints, not by chemical structure, taxonomic origin, or receptor identity. Its validity requires prospective testing of three predictions: synergistic efficacy, functional redundancy, and endpoint specificity (see Section 5.2). The dashed outline indicates its status as a hypothesis-generating framework, not an established therapeutic principle.

### 3.1 The pathogenic arm: LPS-TLR4-LPC axis

Among the many bacterial constituents that leak across a compromised gut barrier, LPS — the major outer-membrane glycolipid of Gram-negative bacteria — is the one most thoroughly implicated in HTG-AP pathogenesis. Under normal conditions, an intact intestinal epithelium restricts LPS entry to negligible levels. But when HTG-induced dysbiosis and barrier erosion coincide, substantial amounts of LPS reach the portal and systemic circulation, a state often called metabolic endotoxemia (37).

Once in the bloodstream, LPS is chaperoned by LPS-binding protein and CD14 to the MD2-Toll-like receptor 4 (TLR4) complex on macrophages, dendritic cells, and, crucially, pancreatic acinar cells. TLR4 engagement triggers both MyD88-dependent and TRIF-dependent signaling cascades, culminating in NF-κB-driven production of TNF-α, IL-1β, and IL-6 (38). Because TLR4 is expressed on acinar cells, this provides a direct molecular conduit through which a gut-derived bacterial signal can influence pancreatic inflammation without requiring intermediary immune cells (14).

The LPS-TLR4-LPC axis is the only axis for which HTG-AP-specific causal evidence currently exists, derived from a multi-omics study in HTG-AP mouse models with FMT and Tlr4 knockout validation (12), complemented by MR studies in human AP broadly (15, 16). A recent multi-omics study by Song et al. (2025) further elucidated this mechanism. In HTG-AP mouse models, HTG-modulated gut microbiota upregulated the host glycerophospholipid pathway and increased lysophosphatidylcholine (LPC) content in both intestinal and pancreatic tissues. Fecal microbiota transplantation from HTG mice transferred this phenotype to recipients, and Tlr4 knockout abrogated the effect entirely. These findings demonstrate that a bacterial structural component (LPS) engages a host innate immune receptor (TLR4) to rewire a host metabolic pathway (glycerophospholipid metabolism), and the resulting metabolite (LPC) directly damages the host organ. Collectively, these findings delineate a mechanistic trajectory spanning luminal dysbiosis, systemic metabolic rewiring, and target-organ damage.

The LPS-TLR4-LPC axis also illustrates the bidirectionality that makes HTG-AP a uniquely self-amplifying condition. HTG initiates the gut dysbiosis that permits LPS translocation. LPS-TLR4 signaling drives LPC accumulation. The resulting systemic inflammation further disrupts the intestinal barrier, facilitating even more LPS entry. Intervening at key nodes within this cycle—whether targeting the microbiota, host receptors, or downstream metabolites—represents a biologically plausible therapeutic strategy.

Before leaving this axis, note that some microbial metabolites can oppose the TLR4 pathway. Bifidobacterium species, particularly B. animalis, produce lactate, which suppresses macrophage-associated inflammation in a TLR4/MyD88- and NLRP3/caspase-1-dependent manner (39). This hints at a broader principle that governs the remaining three axes: specific microbial metabolites, acting through defined host receptors, actively restrain the inflammatory cascades that LPS and LPC ignite. The clinical challenge is that in HTG-AP, the bacteria that produce these protective metabolites are precisely the ones that become depleted.

### 3.2 The counter-regulatory SCFA-GPR43/HDAC axis

Whereas the LPS-TLR4-LPC axis initiates pro-inflammatory cascades, the depletion of SCFA signaling removes a critical host counter-regulatory mechanism. SCFAs, predominantly acetate (C2), propionate (C3), and butyrate (C4), are the most abundant products of bacterial fermentation of dietary fiber in the colon. They are generated by specific taxa within the Firmicutes phylum, most notably Faecalibacterium prausnitzii and various Lactobacillus species (17, 40). In HTG-AP, these SCFA producers are among the most consistently depleted symbionts, and the functional consequence-a steep drop in luminal and circulating SCFA concentrations- has been directly correlated with disease severity.

The evidence for the SCFA axis in AP derives primarily from human AP cohorts of mixed etiologies (41) and from non-HTG AP mouse models (22, 42). Yazici et al. reported that serum acetate and H2S concentrations were significantly higher in AP patients compared with controls (P < 0.001 and P = 0.043, respectively), highlighting the metabolic perturbations in the gut–pancreas axis (41). Whether the same magnitude of SCFA depletion occurs specifically in HTG-AP, and whether the GPR43-dependent protection operates identically in the HTG metabolic milieu, remains to be directly tested. A complementary study by Wang et al. (2025) further demonstrated that SCFA-producing genera show stage-specific depletion patterns, with the most pronounced losses occurring during the early severe phase (43).

SCFAs protect the host through two complementary molecular mechanisms. The first is rapid and receptor-mediated. Acetate, the most abundant SCFA, binds to GPR43 (also known as FFAR2) on intestinal epithelial cells, macrophages, and neutrophils. Guan et al. (2026) provided causal evidence for this axis in AP by showing that Bifidobacterium pseudolongum-derived acetate suppresses M1 macrophage polarization through GPR43-mediated inhibition of NF-κB signaling (22). In Gpr43 knockout mice, the protective effects of both acetate and B. pseudolongum were abolished, establishing that this receptor is necessary for microbial protection. The same group had previously shown that acetate-GPR43 signaling also suppresses NLRP3 inflammasome-driven macrophage pyroptosis, suggesting that this single receptor–ligand pair regulates both the polarization and the death fate of macrophages (42).

The second mechanism is slower but longer-lasting: epigenetic modulation through histone deacetylase (HDAC) inhibition. Butyrate, the most potent HDAC inhibitor among the three major SCFAs, alters histone acetylation at promoter regions of pro-inflammatory genes, leading to sustained transcriptional suppression of cytokines and enhancement of anti-inflammatory mediators (44); by contrast, acetate has been shown to exert its effects through NLRP3 inflammasome modulation rather than HDAC inhibition (45). A 2024 study demonstrated that dietary inulin ameliorates obesity-induced severe AP through the gut–pancreas axis, and that the protective effects of the resulting SCFA mixture could be mimicked by the pharmacological HDAC3 inhibitor RGFP966 (23).

GPR43 ligation and HDAC inhibition exert synergistic protective effects through distinct timelines. While transmembrane receptor activation provides immediate inhibition of acute inflammation, butyrate-mediated histone acetylation regulates sustained anti-inflammatory gene transcription during later stages of AP progression. The collapse of SCFA-producing consortia in HTG-AP thus strips the host of both rapid and sustained counter-regulatory capacities.

These therapeutic strategies, however, must be clinical-context-specific. Preclinical discordance suggests that indiscriminately elevating systemic SCFAs can trigger off-target pro-inflammatory signals if the hyper-acute mucosal environment is already necrotic. This functional non-linearity dictates that SCFA titration cannot be treated as a one-size-fits-all substitution therapy. Rather, it demands precise mapping of the host’s real-time receptor expression profiles and the prevailing inflammatory kinetic window.

### 3.3 The tryptophan-AhR pathway in barrier maintenance

Alongside the SCFA deficit, a second protective metabolic pathway is eroded in HTG-AP: the microbial metabolism of dietary tryptophan to AhR ligands. AhR is a ligand-activated transcription factor that serves as a master regulator of intestinal immunity and barrier function. Upon activation, it translocates to the nucleus and drives expression of target genes including CYP1A1 (xenobiotic metabolism), IL-22 (epithelial regeneration and antimicrobial defense), and mucins (barrier fortification) (21, 46).

The connection to HTG-AP emerged from integrated microbiota–metabolome studies. Chen et al. (2025) employed 16S rRNA gene amplicon sequencing and untargeted metabolomics to investigate the therapeutic mechanism of rhubarb polysaccharides in an HTG-AP mouse model (18). The polysaccharide, a 26 kDa glucose-arabinose polymer, reduced pancreatic injury, improved intestinal histopathology, upregulated tight junction proteins ZO-1 and occludin, and reshaped the gut microbiota by enriching Lactobacillus and Akkermansia while reducing Lachnoclostridium and Desulfovibrio. Untargeted metabolomics identified tryptophan metabolism as the most significantly altered pathway, and integrated analysis revealed strong microbiota–metabolite correlations. The tryptophan-AhR axis has been mechanistically validated in AP mouse models (24) and in HTG-AP mouse models through polysaccharide intervention studies (18); however, the norharman-Rftn1 pathway (24) was established in non-HTG AP models. Direct evidence in HTG-AP patients is currently lacking.

The molecular link between specific tryptophan metabolites and AP protection was elucidated by Zhou et al. (2023), who identified norharman, a β-carboline compound produced by cultivated Lactobacillus species, as an HDAC antagonist (24). AP induced a collapse of Lactobacillus-mediated tryptophan metabolism, reducing norharman availability. Norharman supplementation restored protection by directly inhibiting HDAC1-4 enzymatic activity, increasing H3K9/14 acetylation at the Rftn1 promoter, promoting Raftlin-1 expression, and thereby suppressing macrophage M1 polarization while preserving lipid raft integrity. Myeloid-specific Rftn1 knockout abolished these effects, confirming the target cell and the effector protein. This finding provides a high-resolution mechanistic link by tracing a single microbial metabolite to a specific epigenetic alteration within a defined immune cell population.

What about functional synergy between butyrate and the tryptophan-AhR axis? Butyrate, as a potent HDAC inhibitor, increases histone acetylation at promoter regions broadly. The AHR gene itself is regulated by histone acetylation. This raises the possibility, not yet tested in AP models, that butyrate-mediated HDAC inhibition directly upregulates AhR expression in intestinal or pancreatic tissues. If confirmed, this would constitute a direct molecular bridge between the two protective axes: SCFA-driven epigenetic changes could set the stage for AhR responsiveness, and the loss of butyrate in HTG-AP would therefore diminish AhR signaling not only by depleting its ligands (via loss of tryptophan-metabolizing bacteria) but also by reducing expression of the receptor itself.

### 3.4 Immunomodulation via the bile acid-FXR/TGR5 axis

The fourth co-metabolic axis is both the most recently recognized and the most intimately tied to the primary metabolic defect in HTG-AP — lipid handling. Bile acids, classically viewed as detergents for lipid emulsification, are now understood as potent signaling molecules that regulate inflammation, barrier function, and metabolic homeostasis along the gut–liver–pancreas axis (11). The critical step that connects gut microbiota to bile acid signaling is microbial biotransformation. Primary bile acids, synthesized from cholesterol in the liver and secreted into the duodenum, are converted by gut bacterial enzymes, bile salt hydrolase (BSH) and 7α-dehydroxylase, into secondary bile acids such as deoxycholic acid (DCA) and lithocholic acid (LCA). This conversion substantially expands the molecular diversity and signaling capacity of the bile acid pool (11).

Secondary bile acids engage two principal host receptors: the nuclear receptor farnesoid X receptor (FXR) and the transmembrane G protein-coupled receptor TGR5. FXR activation broadly suppresses NF-κB signaling and pro-inflammatory cytokine production, while TGR5 signaling inhibits NLRP3 inflammasome assembly through the cAMP-PKA pathway and promotes regulatory T cell differentiation (27, 47). Conjugated bile acids have been shown to alleviate AP by inhibiting TGR5 and NLRP3-mediated inflammation (27), and LCA can additionally activate the vitamin D receptor to enhance intestinal barrier function (27).

In HTG-AP, the logic connecting this axis to disease is twofold. For the bile acid axis, direct evidence in AP comes from non-HTG mouse models (25–27). Song et al. (14) provided functional prediction that HTG alters the primary bile acid pool and suppresses BSH-active bacteria, but direct quantification of secondary bile acid depletion in HTG-AP patients has not been reported. First, the HTG state itself alters bile acid synthesis rates and pool composition, establishing a lipotoxic luminal milieu that may directly stress the intestinal epithelium (11). Second, the dysbiosis that accompanies HTG-AP depletes BSH-active and 7α-dehydroxylase-expressing bacteria, disrupting the conversion of primary to secondary bile acids and diminishing FXR/TGR5 occupancy. The predicted outcome is a loss of tonic anti-inflammatory signaling, leaving NLRP3 inflammasome activation and NF-κB-driven cytokine production less restrained.

Direct evidence for this axis in AP has come from multiple laboratories. Wan et al. (2020) demonstrated that bile acid supplementation improves murine pancreatitis in a microbiota-dependent manner (25). More recently, Gu et al. (2026) showed that Odoribacter laneus, a bacterium involved in bile acid metabolism, protects the intestinal barrier in AP specifically through the bile acid-FXR axis, and that this protective effect is transferable via fecal microbiota transplantation (26). Notably, O. laneus does not produce SCFAs directly; its protective mechanism is attributed to bile acid metabolism, underscoring the functional specialization that exists within the gut ecosystem and the need to target multiple metabolic outputs simultaneously.

### 3.5 Network logic and cross-talk of co-metabolic axes

Synthesizing these different pathways reveals that these four metabolic circuits do not function as parallel, isolated tracks. Rather, they form an integrated, self-compensating regulatory network. The LPS-TLR4-LPC axis constitutes the primary pathogenic circuit, driving both local pancreatic injury and systemic inflammation. The three protective axes, SCFA-GPR43/HDAC, tryptophan-AhR, and bile acid-FXR/TGR5, normally restrain this circuit at multiple nodes. They maintain barrier integrity, suppress macrophage M1 polarization, inhibit NLRP3 inflammasome activation, and promote regulatory T cell responses. HTG-AP represents the collapse of this restraint system.

What has been less appreciated is that these axes are not functionally independent. Several molecular connections are plausible and in some cases experimentally supported. Butyrate, through HDAC inhibition, may upregulate AhR expression, linking the SCFA axis to tryptophan responsiveness. AhR activation can modulate the threshold at which TLR4 signaling triggers a full inflammatory response, creating a functional coupling between Axes 2 and 1. Butyrate can also serve as a cross-feeding substrate that supports the colonization of tryptophan-metabolizing bacteria, linking Axes 2 and 3 through an ecological route. Secondary bile acids signal through FXR and TGR5 in both the intestine and the liver, meaning that the bile acid axis can modulate the metabolic context in which the other three axes operate.

Viewing HTG-AP as a functionally coupled network rather than a collection of independent pathways has direct implications for therapy. Single-target interventions, such as supplementing one SCFA, one AhR ligand, or one bile acid species, may encounter compensation from other nodes in the network. This could partially explain why clinical trials of single microbial metabolites have yielded inconsistent results. Multi-axis coordinated strategies that simultaneously restore SCFA production, tryptophan metabolism, bile acid conversion, and barrier integrity may prove more effective precisely because they address the network, not just a node.

## 4 From correlation to causality: evaluating the evidence

Correlation does not equal causation. In a disease as acute and multifactorial as pancreatitis, the risk of mistaking epiphenomena for drivers is substantial. To gauge how far the field has moved beyond descriptive association, we evaluate the evidence against the Bradford Hill criteria — a time-tested framework for judging whether an observed exposure–outcome relationship is likely to be causal. In 1965, Sir Austin Bradford Hill published nine “viewpoints” to help determine whether observed epidemiologic associations are causal. Hill explicitly cautioned that these were not rigid criteria and that “cause-effect decisions cannot be based on a set of rules”; rather, they are aspects to consider collectively, with no hierarchy of importance among them. In this section, we apply these viewpoints to appraise the evidence for a causal role of gut microbiota–host co-metabolism in HTG-AP severity.

### 4.1 Consistency of associations across populations

Strength of association refers to the magnitude of the effect size—the larger the association, the less likely it is to be explained entirely by confounding or bias. Mendelian randomization (MR) studies, which are less susceptible to confounding and reverse causation than conventional observational designs, have identified several gut microbial taxa with statistically significant associations with AP risk. He et al. (2024) reported that genetically predicted abundance of Bacteroidales (OR 1.41, 95% CI 1.057–1.885), the Eubacterium fissicatena group (OR 1.24, 95% CI 1.045–1.470), and Coprococcus3 (OR 1.48, 95% CI 1.049–2.090) was associated with increased AP risk, while Prevotella9 (OR 0.82), Ruminococcaceae UCG004 (OR 0.76), and Ruminiclostridium6 (OR 0.70) showed protective associations (15). A separate MR analysis by Zhao et al. (2025) confirmed inverse causal relationships for Enterococcus B and Faecalicatena torques (48).

These effect sizes are modest (OR range approximately 0.70–1.48), and Hill himself noted that “a small association does not mean there is not a causal effect.” Verdict: Strength of association is partially met—the observed effects are statistically robust and directionally consistent, but the modest magnitude limits the strength of the causal inference from this criterion alone.

Consistency refers to the reproducibility of an association across different populations, study designs, and settings—the more consistently an association is observed, the less likely it is to be a spurious finding. A 2025 meta-analysis integrating MR data from ten studies across European and East Asian cohorts found that the protective role of Odoribacter and the risk-conferring roles of Olsenella and Clostridium leptum were stable across ancestries (19). This cross-population reproducibility argues against these associations being artifacts of a particular genetic background or dietary pattern.

Verdict: Consistency is met—the protective and risk-conferring signals for specific taxa have been replicated across diverse populations and independent MR studies.

### 4.2 Temporal sequence and dose-response relationships

Establishing temporality (that the exposure precedes the outcome) is notoriously difficult in human AP studies because patients present after disease onset. Here, animal models fill the gap. Pre-existing HTG has been shown to alter gut microbiota composition before AP induction, and this altered microbiota predisposes to more severe pancreatic injury (14, 28) — a sequence consistent with the microbiota change being upstream of severity rather than merely reflecting it.

Verdict: Temporality is met in animal models; human data are lacking.

A biological gradient, where greater exposure leads to a greater effect, adds further weight. Clinical studies have demonstrated that the degree of gut microbiota alteration at hospital admission correlates with subsequent disease severity and prognosis (31), and the reduction in SCFA-producing capacity has been associated with disease severity in multiple cohorts (41). This dose–response pattern aligns with the prediction that progressively deeper loss of protective microbial function translates into progressively more severe clinical courses.

Verdict: Biological gradient is partially met—correlative evidence exists in humans, but formal dose–response quantification across the full spectrum of HTG-AP severity awaits prospective studies.

### 4.3 Experimental evidence

Among the Bradford Hill viewpoints, experimental evidence carries substantial weight. Fecal microbiota transplantation (FMT) from HTG-AP mice into antibiotic-pretreated recipients transfers the susceptibility phenotype, and Tlr4 knockout abrogates this effect (14). This result simultaneously demonstrates causality, identifies a specific host receptor as the mechanistic conduit, and establishes microbiota-dependence. The combination of FMT transfer plus host gene knockout represents a particularly rigorous experimental design. Strain-level experiments further support causality: administration of Allobaculum mucilyticum exacerbates HTG-AP severity, while Akkermansia muciniphila alleviates it (28); Bifidobacterium pseudolongum-derived acetate protects against AP through GPR43, with protection abolished in Gpr43 knockout mice (22).

Verdict: Experimental evidence is met—FMT, gene-knockout, and strain-specific intervention studies in animal models collectively support a causal role.

### 4.4 Specificity

Specificity refers to the idea that a particular exposure leads to a specific outcome with no other likely explanation. This criterion is the most criticized of Hill’s viewpoints—it rests on a “one-cause, one-effect” model of disease causation that is rarely applicable in complex, multifactorial diseases. For a pleiotropic exposure such as gut microbial community composition, which influences multiple host physiological systems simultaneously, specificity is not expected to hold.

Indeed, the taxa identified in MR studies (e.g., Odoribacter, Olsenella) have been implicated in other inflammatory and metabolic conditions, reflecting their pleiotropic roles rather than AP-specific effects.

Verdict: Specificity is not applicable to the gut microbiota–host co-metabolism framework. The absence of specificity does not weaken the overall causal case, because the multi-target nature of microbial metabolites is mechanistically expected rather than anomalous. The causal argument rests instead on the convergence of evidence from strength, consistency, temporality, biological gradient, experiment, plausibility, coherence, and analogy.

### 4.5 Plausibility, coherence, and analogy

Plausibility refers to the biological reasonableness of the proposed causal relationship. The four co-metabolic axes outlined in Section 3—LPS-TLR4-LPC, SCFA-GPR43/HDAC, Trp-AhR, and BA-FXR/TGR—provide a coherent mechanistic framework linking gut microbial metabolites to pancreatic inflammation. Each axis has been validated through defined receptor–ligand interactions (TLR4, GPR43, AhR, FXR/TGR5) in relevant experimental systems (12, 16, 21, 23, 24, 41).

Verdict: Plausibility is met.

Coherence requires that the proposed causal relationship be compatible with existing knowledge across multiple lines of evidence. The co-metabolic framework integrates findings from human MR studies (13, 14, 17), animal models (12, 21, 41), and mechanistic biochemistry (16, 23, 25) into a coherent narrative: HTG creates a permissive microbial milieu; dysbiosis erodes the gut barrier; microbial metabolites enter the circulation; and host receptors translate these signals into pancreatic inflammation or protection. The convergence of epidemiological, experimental, and mechanistic evidence supports coherence.

Verdict: Coherence is met.

Analogy considers whether similar causal relationships have been established in related contexts. The gut–pancreas axis in HTG-AP is analogous to the well-established gut–liver axis in metabolic dysfunction-associated steatotic liver disease (MASLD) and the gut–brain axis in neurodegenerative disorders, where microbial metabolites modulate host inflammation and organ function through defined signaling pathways. Additionally, the role of the gut microbiota in other inflammatory conditions (e.g., inflammatory bowel disease, rheumatoid arthritis) provides analogous precedents for microbial influence on systemic inflammation.

Verdict: Analogy is met.

### 4.6 Mediation analysis and cumulative assessment

Further mechanistic support for this co-metabolic relationship emerges from MR mediation analyses. A 2025 bidirectional two-sample MR study by Zeng et al., spanning 473 gut microbiota taxa, 233 circulating metabolites, and 731 immune cell subtypes, found that circulating metabolites—not immune cells—are the key mediators of microbiota effects on pancreatitis risk, with 24 distinct mediation pathways identified (16). A complementary MR analysis further showed that gut microbiota influence AP through inflammatory proteins, adding another mechanistic layer (49).

These mediation results are important because they specify where in the causal chain the critical molecular events reside. Rather than the microbiota influencing AP through a diffuse, uncharacterized route, the evidence points to a defined sequence: microbial composition shapes the circulating metabolome, and specific metabolites engage host receptors to modulate inflammatory tone and barrier function.

#### 4.6.1 Cumulative assessment: a Bradford Hill viewpoint matrix

Assessed against the Bradford Hill viewpoints, the case for a causal role of gut microbiota-host co-metabolism in HTG-AP severity rests on convergent evidence from multiple independent study designs. The matrix below summarizes the verdict for each viewpoint:

**Table:** 

Viewpoint	Verdict	Key supporting evidence
Strength	Partially met	MR: OR 0.70–1.48 for specific taxa (15, 19, 48)
Consistency	Met	Cross-population replication in European and East Asian cohorts (19)
Temporality	Met (animals); not yet (humans)	HTG alters microbiota before AP induction in mice (14, 28)
Biological gradient	Partially met	Microbiota alteration/SCFA reduction correlate with severity (31, 41)
Experimental evidence	Met	FMT transfers susceptibility; gene knockout abrogates effects (14, 22, 28)
Specificity	Not applicable	Pleiotropic community composition; one-cause-one-effect model inapplicable
Plausibility	Met	Four defined receptor–ligand axes (TLR4, GPR43, AhR, FXR/TGR5) (14, 18, 22, 24, 26, 28)
Coherence	Met	Convergence of MR, animal, and mechanistic evidence
Analogy	Met	Gut–liver, gut–brain axes provide analogous precedents

A caveat to this cumulative assessment: with the exception of the LPS-TLR4-LPC axis (14) and limited HTG-AP model data (18, 28, 35), the evidence base derives predominantly from AP broadly. The Bradford Hill appraisal thus supports a causal role of gut microbiota–host co-metabolism in AP broadly, with HTG-AP-specific confirmation currently established for only one axis.

None of these viewpoints alone provides an absolute “smoking gun,” but the cumulative picture—with multiple lines of evidence converging on the same conclusion—makes it increasingly difficult to dismiss the observed microbiota–AP associations as epiphenomena. As Hill himself emphasized, causation is a matter of judgment based on the totality of evidence, not a checklist to be mechanically satisfied.

At the same time, this appraisal clarifies what remains to be demonstrated. The effect sizes identified by MR are modest (strength only partially met); temporality in humans has not been established; biological gradient requires formal dose–response quantification; and the transferability of animal experimental data to the clinical setting—where patients are older, genetically heterogeneous, and treated with antibiotics and supportive care—has not been tested (**Table 1**). These gaps do not undermine the causal inference; they define the translational agenda.

## 5 Translating co-metabolism into precision intervention

Given that HTG-AP clinical severity is dictated by the kinetic balance between pathogenic cascades and counter-regulatory microbial signals, successful therapeutic intervention hinges on breaking this pathological equilibrium. However, transitioning from the conceptual simplicity of ‘suppressing pathogens while restoring symbionts’ to clinical efficacy requires navigating the redundant and highly compensated network cross-talk characterising the gut-pancreas circuit. Because the four axes intersect, interventions that target a single node — supplementing one short-chain fatty acid (SCFA), one aryl hydrocarbon receptor (AhR) ligand, or one bile acid species — may encounter compensation from other nodes in the network. This could partially explain why clinical trials of single microbial metabolites have produced inconsistent results. Effective microbiota-targeted therapy for HTG-AP will likely need to be multi-axis by design. The strategies discussed in this section range from experimentally validated interventions (**Table 1**) to conceptual proposals that remain to be tested. We emphasize that Sections 5.2–5.5 contain proposals and hypotheses, not established clinical guidelines. The guild concept (Section 5.2), the stratification logic (Section 5.5), and the phase-specific treatment algorithm (**Figure 2**) are presented as frameworks to guide future research and trial design, not as evidence-based clinical recommendations.

**Figure 2:**
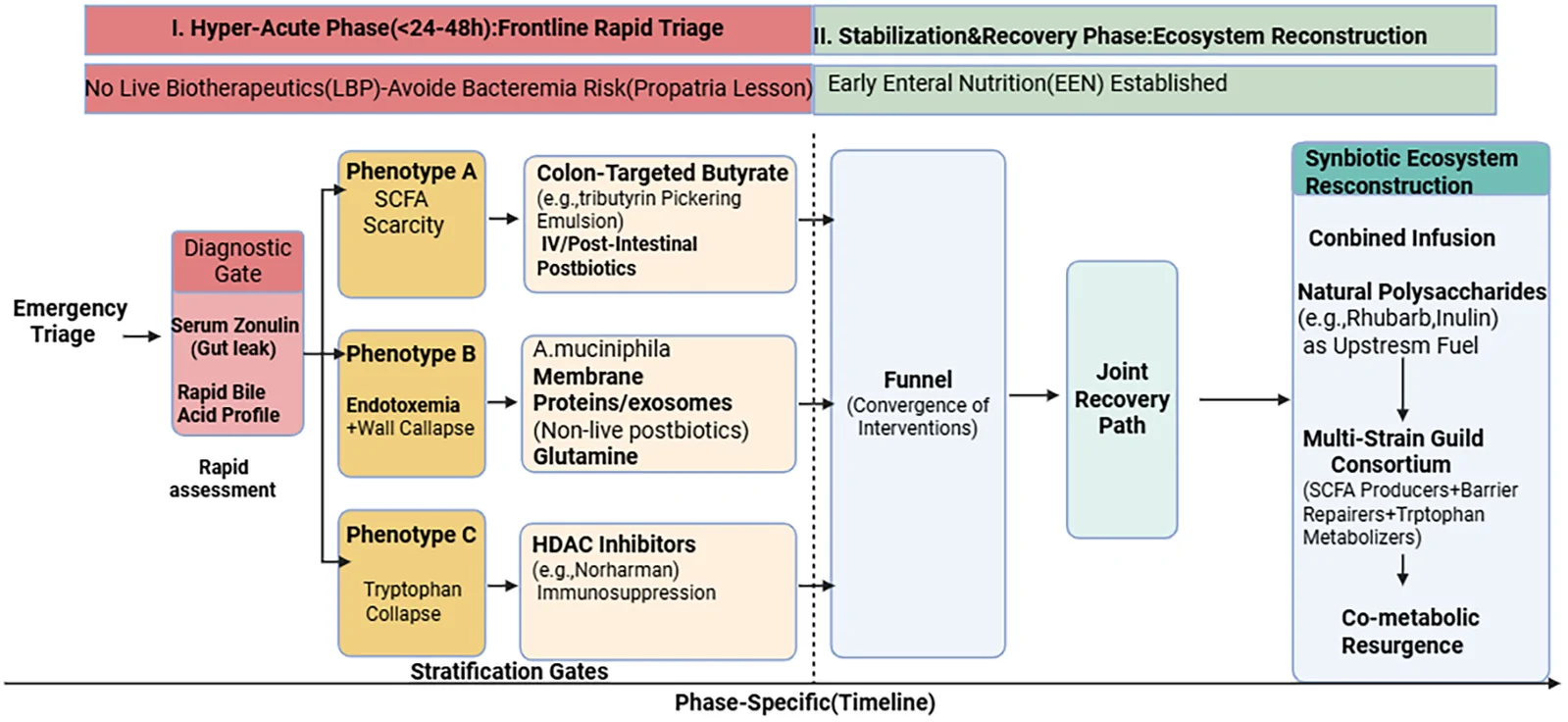
Conceptual translational architecture: a proposed framework for hyper-acute co-metabolic stratification and phase-specific precision intervention.

Before surveying the specific strategies under investigation, we introduce a pragmatic design concept that has guided our thinking. In ecology, a “guild” refers to a group of species that exploit the same resource in a similar manner, regardless of their phylogenetic relatedness (48). Translating this to the metabolite level, we use the term functional metabolite guild to denote a set of microbial-derived metabolites that converge on a common host-protective function, such as maintaining barrier integrity, suppressing NLRP3 inflammasome activation, or promoting regulatory T cell responses. Underpinned by network biology, the simultaneous replenishment of an entire metabolite cluster may exert synergistic efficacy superior to isolated monotherapies, provided these molecules converge on shared protective endpoints. Three such guilds are immediately relevant to HTG-AP, each defined by convergence on a distinct host-protective endpoint rather than by chemical similarity or taxonomic origin: The SCFA guild (acetate, propionate, butyrate) converges on barrier fortification and macrophage de-priming through dual mechanisms—GPR43-mediated rapid signaling and HDAC-dependent epigenetic regulation. The AhR ligand guild (indole-3-aldehyde, indole-3-acetic acid, indole-3-propionic acid, norharman) converges on mucin expression and regulatory T cell differentiation through aryl hydrocarbon receptor activation. The bile acid guild (lithocholic acid, deoxycholic acid) converges on NLRP3 inflammasome suppression through FXR and TGR5 activation.

Metabolites within each guild share a functional endpoint (the “what” of host protection), not necessarily a common biosynthetic pathway, chemical scaffold, or producing organism. This endpoint-centric definition is what distinguishes a guild from a conventional metabolite class. The guild framework generates three experimentally testable predictions. First, synergistic efficacy: simultaneous administration of multiple metabolites within the same guild should produce at least additive—and ideally synergistic—protective effects on the shared endpoint, compared with equimolar doses of any single guild member. Second, functional redundancy: depletion of any single guild member should be compensable by other members of the same guild, such that the net host-protective effect is preserved as long as total guild metabolite concentration remains above a threshold. Third, endpoint specificity: metabolites within a guild should not be interchangeable with metabolites from a different guild targeting a different endpoint—i.e., guild assignments should predict endpoint-specific effects, not promiscuous effects across all protective outcomes. Failure of any of these predictions would falsify the guild framework or require its redefinition. Currently, this guild-based paradigm serves as a mechanistic heuristic for postbiotic formulation; robust empirical comparisons between single-metabolite and guild-based interventions in pancreatitis models remain an active area of investigation.

In the discussion that follows, we use the guild concept as an organizing lens (**Figure 1**) (50). For each intervention category, we ask whether it addresses a single guild, spans multiple guilds, or operates upstream by restoring the ecological conditions that make guild function possible.

### 5.1 Transition to strain-specific probiotics

The era of undefined multi-strain probiotic mixtures is giving way to mechanistically characterized, single-strain interventions. Several candidates have accumulated evidence directly relevant to the co-metabolic deficits described earlier.

Akkermansia muciniphila targets the mucus barrier erosion that is central to HTG-AP pathogenesis. Yang et al. (2025) demonstrated that A. muciniphila supplementation restored mucus layer thickness, reduced intestinal pro-inflammatory macrophage polarization, and alleviated disease severity in HTG-AP mice (28). The protective effects of this bacterium appear to be mediated by specific outer membrane proteins: Amuc_1100 pretreatment alleviates AP by regulating gut microbiota composition and inhibiting inflammatory infiltration (51); Amuc_1409 acts through a Ube2k-Foxp3 axis in regulatory T cells (52); and Amuc_1098 signals via TLR2 to remodel glycerophospholipid metabolism (53). A clinical trial evaluating A. muciniphila PROBIO in HTG-AP patients during the recovery phase is currently recruiting (54, 55).

Faecalibacterium prausnitzii, the most abundant butyrate-producing bacterium in the healthy gut and one of the most consistently depleted symbionts in HTG-AP (31), addresses the SCFA deficit directly. Beyond butyrate, it produces a microbial anti-inflammatory molecule that inhibits NF-κB independently of its fermentation product (20). A 2025 study demonstrated that F. prausnitzii alleviates experimental recurrent AP by producing oleic acid, which regulates MAPK/NF-κB signaling and rebalances the Th17/Treg ratio (29).

Bifidobacterium pseudolongum has been validated specifically through the acetate-GPR43 axis. Guan et al. (2026) showed that B. pseudolongum supplementation attenuates AP through GPR43-mediated suppression of M1 macrophage polarization, with the protection abolished by Gpr43 knockout (22). This validation was performed in a preventive administration model; whether post-onset administration achieves comparable efficacy remains to be determined.

Lactobacillus species offer broader appeal because they simultaneously contribute to multiple protective guilds: producing SCFAs, generating AhR ligands from tryptophan (including norharman (24)), and competitively excluding Gram-negative pathobionts. L. paracasei LP18 ameliorated severe AP through microbiota-mediated regulation of butyrate metabolism (56), and Lactobacillus salivarius Li01 alleviates AP by enriching gut Paramuribaculum and modulating steroid hormone metabolites to suppress pancreatic TNF signaling (57). Clinical trials of L. reuteri (56) and a Bifidobacterium triple viable probiotic (58) are ongoing, alongside an FMT trial for recurrent HTG-AP (59).

Viewed through the guild lens, these strain-specific interventions map onto distinct co-metabolic deficits. A. muciniphila restores the ecological niche on which protective guilds depend — a damaged mucus layer cannot sustain the bacteria that produce guild metabolites, regardless of how many are administered. F. prausnitzii and B. pseudolongum primarily reinforce the SCFA guild. Certain Lactobacillus species have the potential to contribute to both the SCFA and AhR ligand guilds simultaneously. This mapping suggests that a multi-strain consortium built around guild complementarity may be a rational next step in formulation design.

### 5.2 Postbiotics and defined metabolite supplementation

Directly administering microbial metabolites circumvents the main challenges of live bacterial therapeutics: viability during storage, engraftment in a disrupted ecosystem, and the risk of bacteremia in patients with severely increased intestinal permeability. SCFAs, particularly butyrate and propionate, are the most advanced candidates, with established roles in improving intestinal barrier homeostasis and dampening NF-κB-driven inflammation (60). A clinical trial evaluating prophylactic tributyrin supplementation—a butyrate prodrug—in acute pancreatitis is currently registered (NCT06147635) (61).

However, the pharmacokinetics of oral butyrate present a practical obstacle. Rapid absorption in the upper gastrointestinal tract limits colonic delivery, and the pungent volatile profile of raw butyrate makes standard oral compliance practically non-viable. Advanced biomaterials, specifically tributyrin-loaded Pickering emulsions engineered within alginate matrices, offer a robust technological escape from upper gastrointestinal degradation (30). Yet the true translational challenge for these bio-formulations lies in the clinical complexities in the ICU. Severe HTG-AP patients routinely suffer from profound paralytic ileus and high intra-abdominal pressure, and delivering structured micro-particles via narrow-bore nasojejunal feeding tubes presents immediate bio-fouling and clogging risks. Future formulation engineering must prioritize fluid-dynamic compatibility with standard enteral infusion pumps before claiming clinical viability.

Among tryptophan metabolites, norharman stands out as a mechanistically validated lead. As an HDAC1-4 antagonist that suppresses macrophage M1 polarization by promoting Rftn1 expression (24), it targets a node, macrophage epigenetic programming, that is implicated in both the SCFA and AhR axes. Dietary tryptophan supplementation combined with Lactobacillus strains capable of converting tryptophan to AhR ligands could represent a synbiotic strategy that simultaneously provides substrate and biocatalyst. Indole-3-propionic acid, which activates both the pregnane X receptor and AhR, is another candidate worth exploring (62).

For the bile acid guild, secondary bile acids such as lithocholic acid and deoxycholic acid can in principle be supplemented directly, although their detergent properties at high concentrations demand careful dosing. An alternative approach is to enhance endogenous secondary bile acid production by co-administering bile salt hydrolase-active probiotics with dietary fiber.

Whether a guild-based formulation — combining acetate, propionate, butyrate, norharman, and a secondary bile acid in a single delivery system — outperforms single-metabolite supplementation remains an open and testable question. Based on the integrated network dynamics, simultaneous modulation of multiple targets (e.g., GPR43, HDACs, AhR, FXR, and TGR5) may yield superior barrier-restoring effects compared to isolated pathway activation, though this hypothesis requires rigorous empirical validation. But the prediction awaits experimental confrontation, and the safety of administering multiple bioactive metabolites simultaneously in the acute phase must be established before efficacy can be tested.

However, the safety profile of live biotherapeutics in predicted severe AP requires critical re-evaluation. The PROPATRIA trial, which investigated a multi-strain probiotic mixture (Ecologic 641) in 296 patients with predicted severe AP, reported that probiotic prophylaxis did not reduce infectious complications and was associated with an increased risk of mortality (16% vs. 6% in placebo; RR 2.53, 95% CI 1.22–5.25) (63). A subsequent intestinal barrier substudy revealed a more nuanced picture: while the probiotic combination reduced bacterial translocation markers overall, it was associated with increased enterocyte damage (I-FABP) and bacterial translocation specifically in patients with organ failure (64). This subgroup-conditional harm signal underscores that live bacteria and the critically ill gut are a combination that demands extreme caution—particularly in patients with established organ failure. Postbiotics, being non-living, may offer a safer entry point for modulating co-metabolic axes during the acute phase, though this remains to be prospectively tested.

### 5.3 Natural polysaccharides as microbiota-targeted modulators

Natural polysaccharides derived from traditional medicinal plants occupy a middle ground between prebiotics and pharmacologically active agents. Because they resist host enzymatic digestion and reach the colon intact, they can serve as fermentation substrates while simultaneously exerting direct effects on the host.

The best-characterized example in HTG-AP is rhubarb polysaccharide, a 26 kDa glucose-arabinose polymer. In an HTG-AP mouse model, rhubarb polysaccharide reduced serum amylase and lipase, improved pancreatic and intestinal histopathology, upregulated tight junction proteins ZO-1 and occludin, and reshaped the gut microbiota by enriching Lactobacillus and Akkermansia while suppressing Lachnoclostridium and Desulfovibrio. Integrated multi-omics pointed to modulation of gut microbiota-mediated tryptophan metabolism as a key mechanism (18). Dietary inulin, another well-studied polysaccharide, ameliorates obesity-induced severe AP through the gut–pancreas axis, with its protective effects mediated by SCFAs acting through HDAC3 inhibition (23).

Polysaccharides offer several practical advantages: they are generally recognized as safe, can be administered orally or via feeding tubes, and their structural complexity may allow them to engage multiple microbial targets simultaneously. From a guild perspective, polysaccharides operate upstream of the guilds themselves: they provide the substrates that resident bacteria need to produce SCFAs, AhR ligands, and secondary bile acids, but they depend on the presence of the relevant bacterial populations to do so. Their main limitation, therefore, is that their effects are contingent on the recipient’s residual microbiota. A polysaccharide that promotes SCFA production requires the presence of SCFA-producing bacteria to work. In patients whose SCFA-producing taxa have been severely depleted, co-administration with the relevant probiotic strains or direct postbiotic supplementation may be necessary to bridge this gap.

### 5.4 FMT and defined microbial consortia

Fecal microbiota transplantation represents the most comprehensive microbiota-modulating intervention available. Animal studies have provided proof-of-concept: FMT from HTG-AP mice transfers the susceptibility phenotype, and FMT from healthy donors can be protective (14). In the clinical setting, however, the barriers are formidable. The therapeutic window in severe AP is narrow, administration via nasojejunal tube in critically ill patients is logistically complex, the risk of transmitting multidrug-resistant organisms is non-trivial in an era of rising antimicrobial resistance, and the lack of standardized donor screening and preparation protocols makes reproducibility across centers difficult. Published FMT data in AP remain sparse, with one study reporting reduced systemic inflammation accompanied by increases in intestinal Bifidobacterium and Faecalibacterium, but inconsistent effects on intra-abdominal pressure and infectious complications (65).

Defined microbial consortia, i.e., rationally designed mixtures of characterized, beneficial strains that collectively restore lost co-metabolic functions, offer a more refined and potentially safer alternative. The discovery that Odoribacter laneus protects the intestinal barrier through the bile acid-FXR axis (26) is particularly instructive: a single strain capable of engaging multiple protective guilds could serve as a chassis for consortium design. An ideal consortium for HTG-AP might include SCFA-producing strains (F. prausnitzii, Lactobacillus spp., B. pseudolongum), a mucin-restoring strain (A. muciniphila), and a tryptophan-metabolizing strain, formulated to collectively address the co-metabolic deficits described earlier. Whether such a consortium restores guild function more effectively than FMT — and with a more favorable safety profile — remains to be determined.

### 5.5 A co-metabolic stratification logic for precision interventions

A recurring challenge in microbiome-targeted trials is inter-individual variability in baseline microbiota composition. The stratification logic outlined below is a proposed conceptual framework for addressing this heterogeneity in future clinical trial design. It has not been prospectively validated and should not be construed as a clinical decision-making tool. The same probiotic or postbiotic may produce divergent outcomes depending on which co-metabolic deficits a given patient actually harbors. As a conceptual framework for addressing this heterogeneity in future trials, we outline a stratification logic built around the four axes (**Figure 2**). Of note, the components of this framework differ in their evidence base: the association between gut microbiota alteration and disease severity (31), norharman protection in AP models (24), and A. muciniphila efficacy (51–53) are evidence-supported; tributyrin emulsions (30) and serum zonulin (66) are supported by non-HTG-AP or non-pancreatic models and require HTG-AP validation; while the multi-strain guild consortium, phenotype–intervention matching, and rapid bile acid panels remain hypothesis-driven proposals.

The underlying premise is that patients can be provisionally classified according to which co-metabolic axis shows the most pronounced deficit, and interventions should be matched accordingly. For instance, a patient with evidence of reduced SCFA-producing capacity—a pattern associated with disease severity in clinical studies (41)—might be assigned to SCFA guild restoration via colon-targeted butyrate plus acetate and propionate precursors. A patient with elevated plasma LPS and enrichment of Gram-negative pathobionts might benefit most from A. muciniphila supplementation combined with barrier-restoring agents such as zinc or glutamine. A patient with globally reduced tryptophan-derived AhR ligands could be directed toward a tryptophan-rich diet plus AhR ligand-producing Lactobacillus strains. A patient with a low secondary-to-primary bile acid ratio might be a candidate for the bile acid guild, delivered either directly or through bile salt hydrolase-active probiotics.

Mixed-deficit phenotypes are expected to be more common than single-axis collapse states, as the four co-metabolic axes are functionally coupled and dysbiosis typically compromises multiple protective functions simultaneously. For patients presenting with mixed deficits (e.g., concurrent SCFA scarcity and endotoxemia), we propose a hierarchical prioritization strategy: the intervention targeting the most severe or most immediately life-threatening deficit should be deployed first, followed by sequential restoration of secondary deficits once the acute phase stabilizes. This sequencing logic—rather than simultaneous multi-guild administration—is proposed to mitigate the risk of overwhelming an already compromised gut barrier and to allow titration of individual guild components. The optimal sequencing and combination strategies remain hypotheses requiring systematic evaluation in dose-finding and sequential-intervention trials.

The emergency setting imposes severe time constraints on this stratification strategy. Given that severe HTG-AP progresses within a 24-48h window, high-throughput metagenomics is clinically impractical. To capture this therapeutic window, we propose a triage architecture pivoting on rapid point-of-care surrogates, such as serum zonulin or lateral-flow bile acid panels. We acknowledge, however, that the readiness of these biomarker gates for clinical deployment varies substantially. Serum zonulin has been correlated with AP severity (67), but assay standardization and prospective stratification validation are lacking. Rapid bile acid panels have been explored in metabolomic profiling (66), but point-of-care formats do not currently exist. These biomarker gates should therefore be viewed as aspirational research tools requiring further development, not as immediately deployable clinical diagnostics. For near-term trials, simpler stratification proxies—such as clinical severity scores (APACHE II, BISAP) or routine laboratory parameters—may be more practical.

Managing patients with mixed deficits, which is likely the most common scenario, adds another layer of complexity. When both LPS-driven inflammation and SCFA deficiency coexist, the priority may need to be neutralizing the systemic inflammatory trigger before attempting ecosystem restoration. In the hyper-acute phase, when intestinal permeability is at its peak and the risk of bacterial translocation is highest, non-living postbiotics and barrier-supportive measures may be safer than live biotherapeutics. As the patient stabilizes and oral or enteral feeding resumes, targeted probiotics or guild-based consortia could be introduced to restore protective metabolite production. These are, at present, hypotheses that require systematic testing in stepwise clinical trials. The stratification logic is offered as a starting point for trial design, not as a guideline for clinical practice.

This figure presents a conceptual framework for trial design and patient stratification. It is intended to guide hypothesis generation for future precision intervention trials, not to serve as a clinical decision-making tool. The framework integrates evidence-supported components (e.g., norharman, A. muciniphila proteins), partially supported components requiring HTG-AP validation (e.g., tributyrin emulsions, serum zonulin), and hypothesis-driven proposals (e.g., multi-strain guild consortium, bile acid panels). See Section 5.5 for detailed evidence classification.

Phase I: Hyper-Acute Phase (< 24–48h) Frontline Rapid Triage: To match the narrow hyper-acute window and bypass high-throughput metagenomic diagnostic lag, serum zonulin (reflecting gut-leak severity) and rapid lateral-flow bile acid profiling serve as immediate frontline diagnostic gates. Given the documented risks of live bacterial translocation in a breached mucosal environment (PROPATRIA trial (63, 64)), live biotherapeutics should not be administered outside well-designed trials with explicit safety monitoring in this phase. Therapeutic delivery shifts exclusively to non-living, fluid-dynamic compatible postbiotics—such as colon-targeted tributyrin Pickering emulsions for Phenotype A (SCFA scarcity), Akkermansia muciniphila outer membrane proteins/exosomes for Phenotype B (Endotoxemia), and pharmacological HDAC inhibitors (e.g., norharman) for Phenotype C (Tryptophan collapse). All independent phenotype vectors converge through an intervention funnel to stabilize the acute targeted-organ insult.

Phase II: Stabilization & Recovery Phase (Ecosystem Reconstruction): Once early enteral nutrition (EEN) is safely established, the therapeutic paradigm pivots toward joint ecosystem reconstruction. Natural polysaccharides (e.g., rhubarb and inulin polymers) are introduced to act as upstream fuel, stimulating and complementing a rationally designed, multi-strain guild consortium (comprising specialized SCFA producers, mucus repairers, and tryptophan metabolizers) to achieve full co-metabolic resurgence and prevent long-term recurrence.

## 6 Translational barriers: what stands between mechanism and bedside

The co-metabolic framework and the intervention strategies it inspires rest on a chain of evidence that is strongest in animal models and Mendelian randomization studies. Translating these insights into clinical practice requires overcoming significant biological and physiological barriers. The guild concept is a heuristic, not a validated therapeutic principle. The stratification logic is a proposal for trial design, not a guideline for clinical practice. They expose gaps in our understanding of how microbial therapeutics behave in the unique physiological context of acute critical illness.

### 6.1 Safety considerations for live biotherapeutics in the permeable gut

Every study demonstrating probiotic efficacy in AP, without exception, has been conducted in animal models. The leap from a controlled murine experiment to a critically ill human patient is larger in AP than in almost any other condition for which microbiome-targeted therapies are being developed. The central problem is intestinal permeability. Severe AP is defined, in part, by the breakdown of the gut barrier that normally sequesters luminal bacteria and their products. Administering live bacteria into a gut whose barrier function is severely compromised introduces a risk, bacteremia from the therapeutic agent itself, that simply does not exist in the healthy volunteers or outpatient cohorts in whom most probiotic safety data have been generated.

The PROPATRIA trial, which tested a multi-strain probiotic mixture (Ecologic 641) in 296 patients with predicted severe AP, reported higher mortality in the probiotic arm (16% vs. 6%; RR 2.53, 95% CI 1.22–5.25) (63). A subsequent intestinal barrier substudy further revealed that the harm was not uniform across all patients: while the probiotic combination reduced bacterial translocation markers overall, it was associated with increased enterocyte damage (I-FABP) and bacterial translocation specifically in patients with organ failure (64). The mechanism of this subgroup-conditional harm remains debated (translocation of the administered strains, modulation of the mucosal immune response in an already hyperinflamed environment, or an interaction with the standard of care at the time), but the signal is difficult to ignore and carries important implications for patient selection in future trials of live biotherapeutics. Two mitigation strategies are frequently proposed: shifting the intervention window to the very early phase, before barrier disruption peaks, or replacing live bacteria with non-living postbiotics. Both have merit, but neither has been prospectively tested in AP. The postbiotic approach is particularly attractive in light of the guild concept outlined earlier, because it allows multi-axis co-metabolic restoration without introducing replication-competent organisms. The tributyrin supplementation trial currently registered (NCT06147635) will provide the first human safety data for this approach (61). Importantly, the PROPATRIA experience (63, 64) serves as a cautionary reminder that the safety of any microbiota-targeted intervention—whether live or non-living—cannot be assumed from preclinical data alone and requires rigorous evaluation in the specific context of the critically ill, permeable gut.

The persistent failure of preclinical interventions to manifest clinical efficacy is not merely a question of sample size; it is a fundamental problem of ‘chronological dislocation.’ Most animal models test prophylactic or hyper-acute interventions, yet human patients typically present in the 12–24 hour post-symptom window—a phase where the ‘first hit’ of lipotoxicity has already triggered irreversible acinar necrosis and systemic priming. We argue that continued reliance on preventive animal protocols risks generating data that may not translate to the clinical setting, where patients present after symptom onset. Future preclinical efforts must mandate ‘delayed-intervention’ models that mimic the necrotic environment of a clinical ward; if a candidate intervention fails to stabilize the gut barrier in a mouse that has already suffered 24 hours of pancreatic insult, its translational potential for the clinical setting is questionable (22, 56).

### 6.2 Interactions with standard clinical care and nutrition

The routine administration of broad-spectrum antibiotics in AP management exerts a profound and largely indiscriminate suppressive effect on the gut microbial community, including the very consortia that maintain barrier integrity. While infection prophylaxis is understandable, the current standard of care often indiscriminately collapses the very consortia that maintain barrier integrity, effectively sabotaging the baseline upon which any precision microbial intervention must be built. We suggest that the next generation of clinical trials must include antibiotic-usage-status as a primary stratification covariate. Without defining the ecological baseline left by antibiotic usage, we are essentially testing microbial therapeutics in a vacuum, which may explain the high noise-to-signal ratio in current FMT and probiotic data.The questions are both straightforward and unanswered. Does antibiotic pre-exposure reduce probiotic engraftment by depleting the niche that the therapeutic strain would occupy? Should probiotic or postbiotic administration be deferred until after antibiotic clearance to avoid direct killing of the administered strains? Can postbiotics partially compensate for antibiotic-induced depletion of the endogenous microbiota by providing the metabolites that the suppressed community can no longer produce?

A notable exception that proves the rule is the demonstration that rifaximin, a non-absorbable antibiotic, reduced pancreatic injury and systemic inflammation in both murine models and patients with predicted severe AP (68). This finding illustrates a broader point: pharmacologically reshaping the gut ecosystem during AP is feasible, but the direction of reshaping matters profoundly. Narrow-spectrum or non-absorbable agents that suppress Gram-negative pathobionts while sparing SCFA producers may be beneficial. Broad-spectrum antibiotics that indiscriminately collapse the community may eliminate precisely the symbionts that probiotics or prebiotics are intended to restore.

A second neglected interaction involves the relationship between acute lipid-lowering, early enteral nutrition (EEN), and microbiota restoration. Current guidelines emphasize rapid triglyceride reduction through fasting, insulin, heparin, or plasma exchange, and the initiation of EEN within 24-48 hours of admission to maintain intestinal mucosal perfusion and barrier integrity (13). These two interventions, lipid lowering and enteral feeding, are pursued in parallel but have not been conceptually integrated with microbiome-targeted strategies. The reasoning is straightforward: high intraluminal lipid concentrations exert selective pressure on microbial communities (11), and attempting to engraft live probiotics into a lipid-rich, inflamed luminal environment may be biologically futile. Rapid lipid lowering may need to physically clear this lipotoxic barrier before ecosystem restoration can begin.

Once lipids are lowered and EEN is initiated, an opportunity emerges that has barely been explored. EEN formulas could be optimized into disease-specific compositions by incorporating prebiotics such as inulin, which has already been shown to ameliorate obesity-induced severe AP through SCFA-mediated HDAC3 inhibition (44). More ambitiously, EEN could serve as a delivery vehicle for functional metabolite guilds; for instance, tributyrin-loaded nanocarriers designed for colonic release (30) could be incorporated into the feeding formula to simultaneously satisfy caloric needs and provide targeted postbiotic support. These are, at present, conceptual possibilities that require formulation development and safety testing before they can enter clinical investigation.

### 6.3 Population heterogeneity and the translational evidence gap

The significant East-West divergence in HTG-AP epidemiology directly impacts the generalizability of microbiome-based interventions (4). Cross-population metagenomic analyses have revealed consistent differences in gut microbial species and pathways between Western and non-Western populations (5), and the Guangdong Gut Microbiome Project has documented substantial intra-regional variation even within a single Chinese province (6). If the baseline abundance of Bifidobacterium pseudolongum, Faecalibacterium prausnitzii, or Akkermansia muciniphila is already high in a given population — or if the dietary substrates that sustain these bacteria differ between regions — then exogenous supplementation may have limited incremental benefit, and efficacy demonstrated in one population may not replicate in another.

Host genetic variation adds a further dimension that has been almost entirely unexplored. East Asian populations carry a distinct spectrum of loss-of-function variants in LPL, APOA5, and GPIHBP1 that affect intravascular triglyceride clearance and may modulate HTG penetrance and AP severity (9). We hypothesize (and the hypothesis remains untested) that carriers of such risk alleles experience a multi-tiered feedback cascade. Impaired triglyceride clearance leads to compensatory hypersecretion of lipids and primary bile acids into the intestinal lumen, which creates selective pressure that suppresses bile salt hydrolase-active commensals, disrupting secondary bile acid biotransformation and lowering the innate immune threshold for pancreatic inflammation. These gene–microbiota interactions, if they exist, would mean that the optimal co-metabolic intervention for an LPL variant carrier may differ from that for a patient whose HTG is purely diet-driven. Developing population-specific, and ultimately genotype-aware, microbiome-based biomarkers should be a research priority as the field moves toward precision intervention.

Finally, significant gaps remain in the current translational evidence chain. The step from “mechanistically plausible in mice” to “clinically usable in patients” contains multiple gaps that no single study has bridged. Ongoing clinical trials of microbiota-targeted interventions in China (54, 58, 59) remain in early exploratory phases, focused on safety and surrogate biomarkers. Published FMT data in AP show inconsistent effects on hard clinical endpoints (65). The SCFA trial (61) will provide much-needed human data, but it is a single study. What the field needs is a sequence of adequately powered, multi-center randomized controlled trials with rigorous endpoint adjudication and pre-specified stratification by the co-metabolic deficits that the intervention is intended to correct. Without such stratification, a genuinely effective guild-based intervention could be diluted to non-significance by patients who lack the deficit it targets — an error that the framework developed in this review is designed to prevent.

## 7 Knowledge gaps, emerging tools, and future directions

The preceding sections have made the case that HTG-AP is best understood as a co-metabolic disorder in which the gut microbiota plays a causal role. They have also, at multiple points, flagged where the evidence thins out — where animal data have not been corroborated in humans, where mechanisms are plausible but untested, and where the tools needed to translate insights into practice do not yet exist. Here we step back and identify the most consequential gaps, together with the emerging technologies that may help close them.

### 7.1 Addressing the human evidence deficit and functional profiling

Almost everything we know about mechanism comes from rodents. Mouse models have been indispensable for establishing causality; the FMT and gene-knockout experiments reviewed earlier could not have been performed in patients. But rodent gut microbiota differ fundamentally from human gut microbiota in composition, in the substrates available for fermentation, and in the baseline immunological tone of the intestinal mucosa. Moreover, the HTG in most mouse models is induced by genetic manipulation or extreme diets that only partially recapitulate the polygenic, lifestyle-driven hypertriglyceridemia seen in patients. The pressing need is not for more mouse experiments but for well-designed human interventional studies that test, under clinically realistic conditions, whether modulating one or more co-metabolic axes alters outcomes.

The few human trials that exist — the rifaximin study (68), the ongoing A. muciniphila PROBIO trial (54), the SCFA trial (61) — are heterogeneous in design, small in scale, and largely focused on surrogate endpoints. A coordinated effort to establish a multi-center platform trial in HTG-AP, in which patients are characterized at baseline by their co-metabolic profile and randomized to guild-matched versus standard interventions, would represent a decisive step beyond the current fragmentary landscape.

Mendelian randomization studies have identified individual taxa with causal associations to AP risk (15, 19, 48), but naming a taxon is not the same as understanding what it does. Functional redundancy, i.e., the fact that phylogenetically unrelated bacteria can perform the same metabolic conversion, means that therapeutically relevant targets may be guilds of metabolically equivalent organisms, not individual species. Moving beyond 16S rRNA gene amplicon sequencing toward metagenomic and metatranscriptomic approaches that capture the functional gene repertoire of the community is essential for identifying which metabolic capacities are actually depleted in a given patient.

The temporal dimension of this functional depletion is almost entirely unexplored. Most clinical studies capture a single fecal or blood sample at hospital admission. We do not know when dysbiosis begins relative to the onset of hypertriglyceridemia, how it evolves during the hours and days after AP onset, or how it responds to standard interventions such as fluid resuscitation, antibiotics, and early enteral nutrition. Longitudinal studies with serial sampling — ideally beginning in the pre-disease state in high-risk individuals — are needed to distinguish cause from consequence and to identify the window during which interventions can still redirect the trajectory.

The guild concept introduced earlier is, at this stage, a hypothesis-generating tool. Testing it will require preclinical studies that compare single-metabolite supplementation against guild-based formulations in the same AP model, measuring not only organ injury and survival but also the molecular signatures of each co-metabolic axis. If the guild hypothesis is correct, the outcome should be not merely additive but synergistic — the combination should outperform the sum of its parts. If it is not, the field will need to rethink whether the network logic of co-metabolism can be reduced to a formulation strategy, or whether a different approach is needed.

### 7.2 Emerging technologies and multi-omics integration

Several emerging technologies are particularly relevant to the co-metabolic framework. One is single-cell and spatial transcriptomics. Applied to the intestine during AP, these methods have already revealed the dynamic reorganization of epithelial and immune cell populations and identified unexpected actors such as mast cells in early gut dysfunction (69–71). Extending these approaches to the pancreas and the mesenteric lymph nodes, tissues that sit at the intersection of the gut barrier and systemic inflammation, could map, at cellular resolution, where and how microbial metabolites exert their effects.

Another promising area is the study of gut microbiota-derived extracellular vesicles. These nanoparticles, which carry bacterial proteins, lipids, and nucleic acids across the intestinal epithelium and into the systemic circulation, have emerged as a distinct mode of microbiota–host communication that bypasses the need for whole bacteria to translocate (46). Whether the cargo of these vesicles differs between mild and severe HTG-AP, and whether engineered vesicles loaded with guild metabolites could serve as a delivery platform, are open questions with therapeutic potential.

A third area worth watching is the gut virome. A 2026 study profiling the gut virome in AP patients reported profound virome remodeling, with reduced viral diversity and distinct community structures correlating with disease severity and etiology (72). Since bacteriophages are major regulators of bacterial community composition and can mediate horizontal gene transfer of metabolic functions, the virome may influence the behavior of the four co-metabolic axes in ways that go beyond the bacterial census alone.

Ultimately, the co-metabolic framework will need to be embedded in a broader systems biology that integrates host genetics, gut metagenomics, metabolomics, and clinical phenotyping. The interplay between host genetic susceptibility, e.g., variants in LPL, APOC2, APOA5, GPIHBP1, and gut microbiota composition is particularly understudied (9) and may explain some of the inter-individual variability that the lipotoxicity model alone cannot account for. Artificial intelligence and machine learning approaches, which have already been applied to metabolomic data for early prediction of severe AP (66), are natural tools for identifying patterns in multi-omics datasets, but they will require cohorts of sufficient size and diversity to produce models that generalize across populations.

A final gap deserves mention. The microbial–host–isozyme concept (the recognition that microbial enzymes can catalyze reactions that parallel or intersect with host metabolic pathways (11)) and the emerging field of gut microbial enzyme-oriented therapy (73) suggest that the most precise interventions of the future may target not the microbes themselves, nor even their metabolites, but the specific enzymatic activities that produce pathogenic signals or degrade protective ones. This is a longer-term prospect, but it follows logically from the co-metabolic premise: if the language of host–microbiota communication is chemical, then the grammar is enzymatic.

## 8 Concluding remarks

The clinical heterogeneity of HTG-AP exposes the fundamental limitations of a purely pancreato-centric lipotoxicity model. The pathology extends far beyond the pancreas. As current evidence indicates, the gut microbiota actively dictates whether localized injury remains contained or progresses to systemic failure. The gut microbiota, shaped by the very hypertriglyceridemia that defines the disease, participates in a chemical conversation with the host that tips the balance between containment and systemic disease.

Evidence supporting the causal nature of this host-microbiota interaction has strengthened considerably over the past five years. Mendelian randomization studies have identified specific taxa and metabolites with directional effects on pancreatitis risk that are consistent across populations. Gnotobiotic experiments have shown that an HTG-conditioned microbiota can transfer susceptibility. Intervention studies have begun to dissect the molecular pathways, including TLR4, GPR43, HDACs, AhR, FXR, and TGR5, through which microbial metabolites either aggravate or restrain pancreatic inflammation. Mediation analyses have confirmed that circulating metabolites are the central conduit through which the microbiota influences the host.

From this body of work, we have extracted four co-metabolic axes, namely LPS-TLR4-LPC, SCFA-GPR43/HDAC, tryptophan-AhR, and bile acid-FXR/TGR5, that together form an integrated signaling network rather than a collection of independent pathways. The cross-talk among these axes carries a therapeutic implication: multi-axis, guild-based interventions that simultaneously restore several protective metabolic outputs may prove more effective than single-metabolite or single-strain approaches. This is the rationale behind the functional metabolite guild concept and the co-metabolic stratification logic outlined in this review.

We have also been at pains to emphasize what has not yet been demonstrated. The guild concept is a heuristic, not a validated therapeutic principle. The stratification logic is a proposal for trial design, not a guideline for clinical practice. The safety of live biotherapeutics in the critically ill gut remains uncertain, and the impact of antibiotics, lipid-lowering protocols, and early enteral nutrition on microbiome-targeted interventions has barely been studied. The East-West disparity in HTG-AP epidemiology, coupled with known differences in gut microbiota composition and host genetics between populations, means that findings from one region cannot simply be assumed to apply elsewhere.

None of this diminishes the central argument. The gut–pancreas co-metabolic axis is transitioning from a mechanistic observation to a viable, if still experimental, therapeutic target. The next five years will determine whether the framework outlined here can withstand prospective testing. Forthcoming clinical data, including the first postbiotic randomized controlled trials, will either validate the guild-based approach or send us back to the drawing board. Either outcome represents progress. Current evidence effectively refutes the view of the gut microbiota as a passive bystander in HTG-AP. Instead, it acts as a critical disease modulator, and deciphering its metabolic output remains a primary challenge for designing future precision interventions.

## References

[1] Ney A Pereira SP . Acute pancreatitis. Medicine. (2024) 52:99–107. doi: 10.1016/j.mpmed.2023.11.00542574925

[2] Xiao AY Tan MLY Wu LM Asrani VM Windsor JA Yadav D . Global incidence and mortality of pancreatic diseases: a systematic review, meta-analysis, and meta-regression of population-based cohort studies. Lancet Gastroenterol Hepatol. (2016) 1:45–55. doi: 10.1016/S2468-1253(16)30004-8 28404111

[3] Liu Y Cheng JP Zhao XL . The effect of serum triglyceride levels and different lipid-lowering methods on the prognosis of hypertriglyceridemic acute pancreatitis: a single-center 12-year retrospective study by propensity score matching. Scandinavian J Gastroenterol. (2024) 59:1–9. doi: 10.1080/00365521.2024.2342406 38625376

[4] Fan Z Zhang Y Li J He WH Bai XY Cai YN . Global burden and characterization of hypertriglyceridemia-induced acute pancreatitis: results from a systematic review and a multi-center cohort study. Sci China Life Sci. (2025) 68:3010–20. doi: 10.1007/s11427-024-2900-6 40550999

[5] Sheng Y Wang J Gao Y Peng YL Li X Huang WH . Combined analysis of cross-population healthy adult human microbiome reveals consistent differences in gut microbial characteristics between Western and non-Western countries. Comput Struct Biotechnol J. (2023) 23:87–95. doi: 10.1016/j.csbj.2023.11.047 38116074 PMC10730331

[6] Chen YR Jing QL Chen FL Zheng H Chen LD Yang ZC . Desulfovibrio is not always associated with adverse health effects in the Guangdong Gut Microbiome Project. PeerJ. (2021) 9:e12033. doi: 10.7717/peerj.12033 34466295 PMC8380029

[7] Adiamah A Psaltis E Crook M Lobo DN . A systematic review of the epidemiology, pathophysiology and current management of hyperlipidaemic pancreatitis. Clin Nutr. (2018) 37:1810–22. doi: 10.1016/j.clnu.2017.09.028 29056284

[8] Lu J Wang Z Mei W Peng KX Zhang L Wang G . A systematic review of the epidemiology and risk factors for severity and recurrence of hypertriglyceridemia-induced acute pancreatitis. BMC Gastroenterol. (2025) 25:374. doi: 10.1186/s12876-025-03954-4 40375154 PMC12082898

[9] Carr RA Rejowski BJ Cote GA Pitt HA Zyromski NJ . Systematic review of hypertriglyceridemia-induced acute pancreatitis: a more virulent etiology? Pancreatology. (2016) 16:469–76. doi: 10.1016/j.pan.2016.02.011 27012480

[10] Banks PA Bollen TL Dervenis C Gooszen HG Johnson CD Sarr MG . Classification of acute pancreatitis--2012: revision of the Atlanta classification and definitions by international consensus. Gut. (2013) 62:102–11. doi: 10.1136/gutjnl-2012-302779 23100216

[11] Song XF Liu Y Fei QM Xu CL Ji FP . Potential influence of gut microbiota on the process of hypertriglyceridemia-aggravated acute pancreatitis. World J Gastroenterol. (2026) 32:114479. doi: 10.3748/wjg.v32.i1.114479 41551527 PMC12809160

[12] Qiu M Zhou X Zippi M Goyal H Basharat Z Jagielski M . Comprehensive review on the pathogenesis of hypertriglyceridaemia-associated acute pancreatitis. Ann Med. (2023) 55:2265939. doi: 10.1080/07853890.2023.2265939 37813108 PMC10563627

[13] Li XY He C Zhu Y Lu NH . Role of gut microbiota on intestinal barrier function in acute pancreatitis. World J Gastroenterol. (2020) 26:2187–93. doi: 10.3748/wjg.v26.i18.2187 32476785 PMC7235204

[14] Song XF Qiao L Dou X Chang JJ Zeng XN Deng TJ . Hypertriglyceridemia-modulated gut microbiota promotes lysophosphatidylcholine generation to aggravate acute pancreatitis in a TLR4-dependent manner. iMeta. (2025) 4:e70003. doi: 10.1002/imt2.70003 40027483 PMC11865321

[15] He L Luo H Li Y Zhang Y Peng L Xu Y . The causal relationship between the gut microbiota and acute pancreatitis: a 2-sample Mendelian randomization study. Med (United States). (2024) 103:e38331. doi: 10.1097/MD.0000000000038331 39259083 PMC11142829

[16] Zeng YZ Ye L Zou WB Xu ZQ . The gut microbiota, circulating metabolites, immune cells, and pancreatitis risk: Evidence from a bidirectional two-sample Mendelian randomization study and mediation analysis. Medicine. (2025) 104:e45216. doi: 10.1097/MD.0000000000045216 41137328 PMC12558190

[17] Guo Q Wu XD . The role of intestinal microbiota and its metabolite short-chain fatty acids in hypertriglyceridemia-associated acute pancreatitis. Front Microbiol. (2025) 16:1667075. doi: 10.3389/fmicb.2025.1667075 41277964 PMC12631465

[18] Chen Y Zhang W Mao Y Hu G Chen XX Zhou YL . Rhubarb polysaccharides alleviate hypertriglyceridemia-induced acute pancreatitis and regulate gut microbiota-mediated tryptophan metabolism. J Funct Foods. (2025) 135:107104. doi: 10.1016/j.jff.2025.10710442574925

[19] Yi A Zhang W Wei AQ Xie GH . Towards microbiota-targeted interventions in acute pancreatitis: Evidence from meta-analysis and Mendelian randomization. FASEB J. (2025) 39:e70938. doi: 10.1096/fj.202501787R 40838520 PMC12368941

[20] Xiong YY Wu D Qian JM . Role of gut microbiota and its metabolites in severe acute pancreatitis-related acute respiratory distress syndrome. J Clin Hepatol. (2021) 37:233–6. doi: 10.3969/j.issn.1001-5256.2021.01.052

[21] Liu Q Ruan KY An ZH Li LY Ding C Xu DC . Updated review of research on the role of the gut microbiota and microbiota-derived metabolites in acute pancreatitis progression and inflammation-targeted therapy. Int J Biol Sci. (2025) 21:1242–58. doi: 10.7150/ijbs.108858 39897025 PMC11781165

[22] Guan LY Li XY He C Zheng P Zou YY Zhu Q . Bifidobacterium pseudolongum-derived acetate attenuates acute pancreatitis through GPR43-mediated suppression of M1 macrophage polarization. Adv Sci. (2026) 13:2198–3844. doi: 10.1002/advs.202517642 41903115 PMC13252603

[23] Li X Zheng P Zou YY Guan LY Li NS Liu JP . Dietary inulin ameliorates obesity-induced severe acute pancreatitis via gut-pancreas axis. Gut Microbes. (2024) 16:2436949. doi: 10.1080/19490976.2024.2436949 39653685 PMC11633219

[24] Zhou Q Tao XF Guo FY Wu Y Deng DW Lv LL . Tryptophan metabolite norharman secreted by cultivated Lactobacillus attenuates acute pancreatitis as an antagonist of histone deacetylases. BMC Med. (2023) 21:329. doi: 10.1186/s12916-023-02997-2 37635214 PMC10463520

[25] Wan YD Zhu RX Pan XT Sun TW . Bile acid supplementation improves murine pancreatitis in association with the gut microbiota. Front Physiol. (2020) 11:650. doi: 10.3389/fphys.2020.00650 32612539 PMC7309677

[26] Gu RR Li JZ Qi JF Sun RN Wu HL Jin B . Odoribacter laneus protects intestinal barrier by bile acid-FXR axis in acute pancreatitis. AMB Express. (2026) 16(1). doi: 10.1186/s13568-026-02071-z 42162499 PMC13365277

[27] Zhang ZY Guo XL Yi Liu JT Gu YY Ji XW Zhu S . Conjugated bile acids alleviate acute pancreatitis through inhibition of TGR5 and NLRP3 mediated inflammation. J Transl Med. (2024) 22:1124. doi: 10.1186/s12967-024-05922-0 39707318 PMC11662532

[28] Yang P Wu MR Liang WJ Sun YD Pan LL Sun J . Allobaculum mucilyticum-mediated gut barrier dysfunction exacerbates the severity of hypertriglyceridemic acute pancreatitis in mice. Antioxidants (Basel). (2025) 14:1284. doi: 10.3390/antiox14111284 41300441 PMC12649440

[29] Qin WF Mei QX Wang GQ Wang RL Huang ZH Fu Y . Faecalibacterium prausnitzii alleviates experimental recurrent acute pancreatitis by producing oleic acid to regulate MAPK/NF-κB signaling and Th17/Treg balance. NPJ Biofilms Microbiomes. (2025) 11:1–18. doi: 10.1038/s41522-025-00845-0 41271774 PMC12660694

[30] Sun GG Li WB Li DH Hu XW Li B McClements DJ . Amelioration of colitis using colonic microbiota-stimulated release of tributyrin from Pickering emulsion-filled alginate beads. Langmuir. (2025) 41:20081–95. doi: 10.1021/acs.langmuir.5c02248 40695562

[31] Hu XM Gong L Zhou RL Han ZY Ji L Zhang Y . Variations in gut microbiome are associated with prognosis of hypertriglyceridemia-associated acute pancreatitis. Biomolecules. (2021) 11:695. doi: 10.3390/biom11050695 34066441 PMC8148198

[32] Zhu Y He C Li XY Cai Y Hu JX Liao YH . Gut microbiota dysbiosis worsens the severity of acute pancreatitis in patients and mice. J Gastroenterol. (2019) 54:347–58. doi: 10.1007/s00535-018-1529-0 30519748

[33] Deng XY Wu XQ Wang RB Qiao XH Cao T Xu Y . Gut microbiota-based biomarkers for precision subtype classification and mechanistic understanding of biliary and hyperlipidemic acute pancreatitis. Front Microbiol. (2025) 16. doi: 10.3389/fmicb.2025.1695811 41341501 PMC12669208

[34] Christoph AH Richard M Kai LA Thomas A Silke C Gabriele C . Gut microbiota predict development of postdischarge diabetes mellitus in acute pancreatitis. Gut. (2026) 75:316–25. doi: 10.1136/gutjnl-2025-336715 41298102 PMC12911579

[35] Huang CL Chen J Wang JJ Zhou H Lu YY Lou LH . Dysbiosis of intestinal microbiota and decreased antimicrobial peptide level in Paneth cells during hypertriglyceridemia-related acute necrotizing pancreatitis in rats. Front Microbiol. (2017) 8:776. doi: 10.3389/fmicb.2017.00776 28522995 PMC5415626

[36] Fu Y Mei QX Yin NM Huang ZH Li BW Luo SZ . Paneth cells protect against acute pancreatitis via modulating gut microbiota dysbiosis. mSystems. (2022) 7:e0150721. doi: 10.1128/msystems.01507-21 35491818 PMC9239092

[37] Su YR Hong YP Mei FC Wang CY Li M Zhou Y . High-fat diet aggravates the intestinal barrier injury via TLR4-RIP3 pathway in a rat model of severe acute pancreatitis. Mediators Inflammation. (2019) 2019:2512687. doi: 10.1155/2019/2512687 31933540 PMC6942875

[38] Zhang XY Yang GD Chen YL Mu Z Zhou HY Zhang LY . Resveratrol pre-treatment alleviated caerulein-induced acute pancreatitis in high-fat diet-feeding mice via suppressing the NF-κB proinflammatory signaling and improving the gut microbiota. BMC Complementary Med Ther. (2022) 22:189. doi: 10.1186/s12906-022-03664-4 35842665 PMC9288014

[39] Li H Xie JY Guo XL Yang GL Cai B Liu JTY . Bifidobacterium spp. and their metabolite lactate protect against acute pancreatitis via inhibition of pancreatic and systemic inflammatory responses. Gut Microbes. (2022) 14:2127456. doi: 10.1080/19490976.2022.2127456 36195972 PMC9542615

[40] Wang ZJ Guo MY Yang S Chen YP Cheng JB Huang ZW . Intestinal microflora and metabolites affect the progression of acute pancreatitis (AP). Gut Pathog. (2024) 16:64. doi: 10.1186/s13099-024-00652-6 39478598 PMC11526647

[41] Yazici C Thaker S Castellanos KK Al Rashdan H Huang Y Sarraf P . Diet, gut microbiome and their end-metabolites associate with acute pancreatitis risk. Clin Trans Gastroenterol. (2023) 14:e00597. doi: 10.14309/ctg.0000000000000597 37162146 PMC10371326

[42] Li TT Deng FY Xiong J Wu FQ Tang SX Zhang YJ . The mechanism of short-chain fatty acid in hypertriglyceridemic pancreatitis. Front Microbiol. (2026) 16:1714013. doi: 10.3389/fmicb.2025.1714013 41710514 PMC12910481

[43] Wang H Chen YM Han Y Mu SC Wei W Lan LL . Comparative study of gut microbiota and metabolite variations between severe and mild acute pancreatitis patients at different stages. Microb Pathogen. (2025) 198:107030. doi: 10.1016/j.micpath.2024.107030 39536839

[44] Deng XY Zhang HL Chen WD Wang YD . Epigenetic orchestrator and drug enhancer: dual roles of butyrate in regulating post-translational modification and optimizing therapeutic delivery. Cell Commun Signaling. (2025) 23:501. doi: 10.1186/s12964-025-02520-5 41267129 PMC12632102

[45] Niu JL Cui MM Yang X Li J Yao YH Guo QH . Microbiota-derived acetate enhances host antiviral response via NLRP3. Nat Commun. (2023) 14:642. doi: 10.1038/s41467-023-36323-4 36746963 PMC9901394

[46] Li YJ Li J Li S Zhou SM Yang JH Xu K . Exploring the gut microbiota's crucial role in acute pancreatitis and the novel therapeutic potential of derived extracellular vesicles. Front Pharmacol. (2024) 15:1437894. doi: 10.3389/fphar.2024.1437894 39130638 PMC11310017

[47] Chen X Chen X Yan D Zhang N Fu W Wu MX . GV-971 prevents severe acute pancreatitis by remodeling the microbiota-metabolic-immune axis. Nat Commun. (2024) 15:1–18. doi: 10.1038/s41467-024-52398-z 39333064 PMC11436807

[48] Zhao ZR Han L Tuerxunbieke B Ming L Ji JM Chen Y . Effects of gut microbiota and metabolites on pancreatitis: a two-sample Mendelian randomization study. J Gastrointestinal Surg. (2025) 29:101885. doi: 10.1097/MD.0000000000045216 39549891

[49] Huang PY Liu Q Zhang TL Yang JF . Gut microbiota influence acute pancreatitis through inflammatory proteins: a Mendelian randomization analysis. Front Cell Infect Microbiol. (2024) 14:1380998. doi: 10.3389/fcimb.2024.1380998 38881734 PMC11176513

[50] Simberloff D Dayan T . The guild concept and the structure of ecological communities. Annu Rev Ecol Systematics. (1991) 22:115–43. doi: 10.1146/annurev.ecolsys.22.1.11541139587

[51] Wang LJ Jin YL Pei WL Li JC Zhang RL Wang JJ . Amuc_1100 pretreatment alleviates acute pancreatitis in a mouse model through regulating gut microbiota and inhibiting inflammatory infiltration. Acta Pharmacol Sin. (2024) 45:570–80. doi: 10.1038/s41401-023-01186-4 38012292 PMC10834448

[52] Xie JY Du LJ Lu YK Guo XL Zhou XY Tong YF . Akkermansia muciniphila alleviates severe acute pancreatitis via Amuc1409-Ube2k-Foxp3 axis in regulatory T cells. Adv Sci. (2025) 12:e04214. doi: 10.1002/advs.202504214 40464424 PMC12376690

[53] Wang LJ Zhang RL Zhao LT Xiao LX Zhang XY Liu LY . The membrane protein Amuc_1098 from Akkermansia muciniphila alleviates acute pancreatitis via TLR2 signaling and glycerophospholipid metabolism remodeling. Nat Commun. (2026) 17(1). doi: 10.1038/s41467-026-71140-5 42000727 PMC13275887

[54] Chinese Clinical Trial Registry . Akkermansia muciniphila PROBIO for lipid-lowering in HTG-AP recovery phase. In: Chictr. China: Chinese Clinical Trial Registry (2025).

[55] Chinese Clinical Trial Registry . Lactobacillus reuteri and its metabolites in post-pancreatitis endocrine function and pancreatic immunity. In: Chictr. China: Chinese Clinical Trial Registry (2025).

[56] Cao JL Song AR Zhang Q Zhang TJ Jia XY Li B . Lactobacillus paracasei LP18 ameliorated inflammation and intestinal barrier dysfunction in severe acute pancreatitis via gut microbiota-mediated regulation of butyrate metabolism. Front Microbiol. (2026) 17:1765127. doi: 10.3389/fmicb.2026.1765127 41767557 PMC12936515

[57] Duan JM Lou J Wang FY Jiang HY Lv LX Fu HW . Multi-omics analysis reveals the potential preventive mechanism of Lactobacillus salivarius Li01 against L-arginine-induced acute pancreatitis in mice. Microb Biotechnol. (2026) 19:e70300. doi: 10.1111/1751-7915.70300 41543515 PMC12810401

[58] Chinese Clinical Trial Registry . Bifidobacterium triple viable probiotic for HTG-AP infectious complications. In: Chictr. China: Chinese Clinical Trial Registry (2025).

[59] Chinese Clinical Trial Registry . Fecal microbiota transplantation for recurrent HTG-AP. In: Chictr. China: Chinese Clinical Trial Registry (2025).

[60] Zhu Y Mei QX Fu Y Zeng Y . Alteration of gut microbiota in acute pancreatitis and associated therapeutic strategies. Biomedicine Pharmacotherapy. (2021) 141:111850. doi: 10.1016/j.biopha.2021.111850 34214727

[61] Pancreatitis - Microbiome as Predictor of Severity II. Clinicaltrials.Gov Identifier: NCT06147635. First Posted: June 17, 2024. Available online at: https://clinicaltrials.gov/ct2/show/NCT06147635 (Accessed June 13, 2026).

[62] Peng RP Song C Gou SQ Liu HY Kang HL Dong YM . Gut Clostridium sporogenes-derived indole propionic acid suppresses osteoclast formation by activating pregnane X receptor. Pharmacol Res. (2024) 202:107121. doi: 10.1016/j.phrs.2024.107121 38431091

[63] Besselink MGH van Santvoort HC Buskens E Boermeester MA van Goor H Timmerman HM . Probiotic prophylaxis in predicted severe acute pancreatitis: a randomised, double-blind, placebo-controlled trial. Lancet. (2008) 371:651–9. doi: 10.1016/S0140-6736(08)60207-X 18279948

[64] Renooij W van Santvoort HC Besselink MG . Intestinal barrier dysfunction in a randomized trial of a specific probiotic composition in acute pancreatitis. Ann Surg. (2009) 250:712–9. doi: 10.1097/SLA.0b013e3181bce5bd 19801929

[65] Cymbal M Chatterjee A Baggott B . Fecal microbiota transplantation: Current evidence and future directions. Cleveland Clinic J Med. (2025) 92:421–8. doi: 10.3949/ccjm.92a.24107 40592538

[66] Deng DW Yuan QH Pan C Chen JH Liu JJ Wei S . Non-invasive liquid biopsy based on metabolomic profiling improves diagnosis and early warning of severe acute pancreatitis. NPJ Digital Med. (2026) 9:1–15. doi: 10.1038/s41746-025-02294-7 41611815 PMC12868633

[67] Avcıoğlu U Eruzun H . Serum zonulin levels as an early biomarker in predicting the severity and complications of acute pancreatitis. Eurasian J Med. (2023) 55:78–82. doi: 10.5152/eurasianjmed.2022.0272 36861872 PMC10081017

[68] Zou YY Yu BJ He C Ding L Xu X Wan JH . Rifaximin reduces gut-derived inflammation in severe acute pancreatitis: an experimental animal model and randomized controlled trial. Microbiol Spectr. (2025) 13:e0129925. doi: 10.1128/spectrum.01299-25 40919785 PMC12502533

[69] Wei ZX Jiang SH Qi XY Cheng YM Liu Q Hou XY . scRNA-seq of the intestine reveals the key role of mast cells in early gut dysfunction associated with acute pancreatitis. World J Gastroenterol. (2025) 31:103094. doi: 10.3748/wjg.v31.i12.103094 40182603 PMC11962851

[70] Li B . From scRNA-seq to therapeutic targets: Unveiling the impact of activated mast cells on intestinal dysfunction in acute pancreatitis. World J Gastroenterol. (2025) 31:107865. doi: 10.3748/wjg.v31.i25.107865 40656612 PMC12243856

[71] Zhang LC Xu CQ Su T Xiao T Yi X Feng YM . Single-nucleus and spatial transcriptomics reveal intestinal cellular heterogeneity, differentiation, and cell communication mechanisms in SAP-induced intestinal injury. Front Immunol. (2026) 17:1719902. doi: 10.3389/fimmu.2026.1719902 41694378 PMC12901460

[72] Liu M Wang LY Liu JJ Yuan Q Zhang YT Wu SY . Gut virome and metabolic associations in patients with acute pancreatitis. mSystems. (2026) 11:e0140025. doi: 10.1128/msystems.01400-25 41789894 PMC13011395

[73] Ding Y Zhang ZW Wang K Jiang CT . The microbiome regulates host metabolic health and diseases through microbial enzymes. Nat Rev Gastroenterol Hepatol. (2026) 23(7):563–84. doi: 10.1038/s41575-026-01195-8 41946922

